# Integration of Gold Nanoparticles into BiVO_4_/WO_3_ Photoanodes via Electrochromic Activation of WO_3_ for Enhanced Photoelectrochemical Water Splitting

**DOI:** 10.1021/acsaem.4c02735

**Published:** 2025-03-28

**Authors:** Ali Can Güler, Milan Masař, Michal Urbánek, Michal Machovský, Mohamed M. Elnagar, Radim Beranek, Ivo Kuřitka

**Affiliations:** † Centre of Polymer Systems, Tomas Bata University in Zlin, Tr. T. Bati 5678, 760 01 Zlin, Czech Republic; ‡ Institute of Electrochemistry, Ulm University, Albert-Einstein-Allee 47, 89081 Ulm, Germany; § Department of Chemistry, Faculty of Technology, Tomas Bata University in Zlín, Vavrečkova 5669, 760 01 Zlín, Czech Republic; ∥ Faculty of Chemistry, Jagiellonian University, ul. Gronostajowa 2, Kraków 30-387, Poland

**Keywords:** bismuth vanadate, tungsten oxide, electrochromism, gold nanoparticles, surface plasmon resonance, ternary junction, photoelectrochemical water splitting

## Abstract

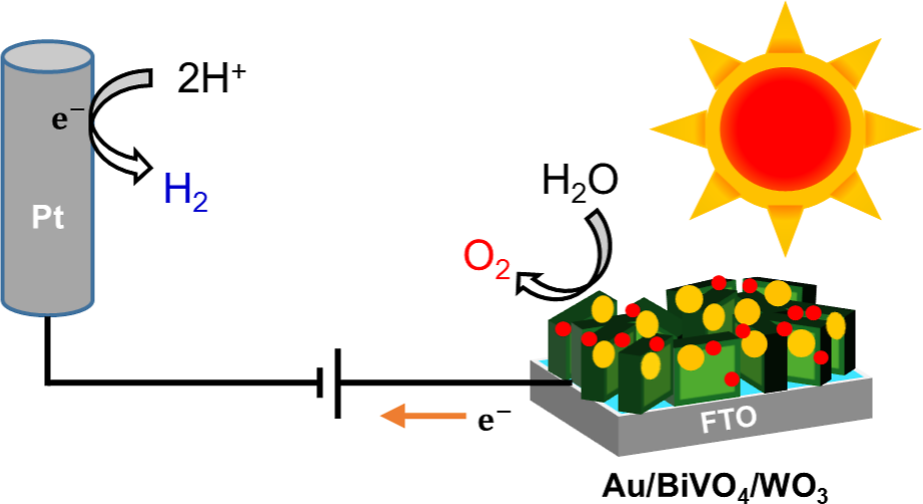

The development of highly efficient photoanodes is crucial for enhancing the energy conversion efficiency in photoelectrochemical water splitting. Herein, we report an innovative approach to fabricating an Au/BiVO_4_/WO_3_ ternary junction that leverages the unique benefits of WO_3_ for efficient electron transport, BiVO_4_ for broadband light absorption, and Au nanoparticles (NPs) for surface plasmon effects. The BiVO_4_/WO_3_ binary junction was constructed by depositing a BiVO_4_ layer onto the surface of the WO_3_ nanobricks via consecutive drop casting. Au NPs were subsequently integrated into the BiVO_4_/WO_3_ structure through electrochromic activation of WO_3_. The optimal BiVO_4_ loading for the highest-performing BiVO_4_/WO_3_ heterostructure and the light intensity dependence of the photocurrent efficiency were also determined. Flat-band potential measurements confirmed an appropriate band alignment that facilitates electron transfer from BiVO_4_ to WO_3_, while work function measurements corroborated the formation of a Schottky barrier between the incorporated Au NPs and BiVO_4_/WO_3_, improving charge separation. The best-performing Au NP-sensitized BiVO_4_/WO_3_ photoanode thin films exhibited a photocurrent density of 0.578 mA cm^–2^ at 1.23 V vs RHE under AM 1.5G (1 sun) illumination and a maximum applied-bias photoconversion efficiency of 0.036% at 1.09 V vs RHE, representing an enhancement factor of 12 and 2.3 compared to those of pristine BiVO_4_ and WO_3_ photoanodes, respectively. This study presents a promising and scalable route for fabricating noble metal-sensitized, metal oxide-based nanocomposite photoanodes for solar water splitting.

## 1 Introduction

Increasing energy demand due to population and industrial growth is rapidly depleting limited resources available worldwide. Most of the global energy demand is met by using fossil fuels due to their high energy density. However, the excessive use of these fuels leads to climate change, as they release significant amounts of greenhouse gases, such as carbon dioxide. Using renewable sources such as solar energy can mitigate the dependence on fossil fuels and reduce greenhouse gas emissions. However, solar energy is an intermittent energy resource and thus cannot provide a consistent supply, matching with our current energy consumption practices. However, free energy can be stored within the chemical bonds of chemical compounds such as hydrogen and controllably released through exothermic reactions without emitting greenhouse gases.^1^

Photoelectrochemical (PEC) water splitting is a promising technology to generate and store green energy, as it directly converts solar energy into chemical energy by splitting water into hydrogen and oxygen. The PEC water-splitting process consists of two half-reactions: the oxygen evolution reaction (OER), occurring through a four-electron process in the photoanode, and the hydrogen evolution reaction (HER), occurring through a two-electron process in the photocathode.^2^ The OER is the key limiting step in the PEC water splitting since it is mechanistically more complex and associated with higher overpotentials than the HER. Therefore, a lot of scientific effort has been devoted to developing more efficient photoanode materials.^3−5^

Tungsten trioxide (WO_3_) is a widely used photoanode material in PEC water splitting and is recognized for its exceptional properties. With a band gap of approximately 2.6 eV, WO_3_ efficiently absorbs visible light, making it suitable for solar energy applications. Its high chemical stability ensures long-term operation, while its excellent electron mobility promotes efficient charge transport. Additionally, the strong oxidizing power of WO_3_, attributed to its optimal valence band (VB) position, significantly enhances its performance in PEC processes.^6,7^ Moreover, WO_3_ can be combined with various semiconductors to further enhance its PEC performance. Commonly used semiconductors include iron oxide (Fe_2_O_3_),^8^ polymeric carbon nitride,^9^ copper oxide (Cu_2_O),^10^ and bismuth vanadate (BiVO_4_).^11,12^ Among these semiconductors, BiVO_4_ stands out as a promising candidate for use as a photosensitizer because it has a narrower band gap (∼2.4 eV) than WO_3_ for extended light absorption and suitable band alignment with WO_3_ for effective charge separation. In our previous paper,^13^ BiVO_4_ nanoparticles (NPs) electrodeposited 3D hierarchal ZnO nanodendrites demonstrated an improved PEC response due to the significantly extended light absorption toward the visible region of the solar spectrum. The synergistic effect of WO_3_ and BiVO_4_ leads to a higher photocurrent compared to that using either material alone, driven by enhanced charge separation and improved utilization of the solar spectrum. Grigioni et al. have optimized the BiVO_4_ layer thickness on WO_3_, identifying a 75 nm thick BiVO_4_ layer as the best-performing photoelectrode.^14^ Furthermore, BiVO_4_ was applied on 1-dimensional WO_3_ nanorods; the resulting BiVO_4_/WO_3_ with optimal thickness produced a photocurrent up to 3.8 mA cm^–2^ at 1.23 V vs reversible hydrogen electrode (RHE) under simulated solar light.^15^ On the basis of the versatility in structural design, Liu et al. have shown that the PEC performance of WO_3_ strongly depends on its different crystal facets owing to different surface energies and electronic structures.^16^ In their research, it was underlined that WO_3_ with (002) facets generated the highest faradaic efficiency on PEC water splitting when preferentially exposed to the (020) BiVO_4_ facet due to a better hole injection efficiency at the interface between the two materials.

To enhance the efficiency of solar water splitting with the BiVO_4_/WO_3_ photoanode, this heterojunction was further integrated with additional materials, including cocatalysts, passivation layers, and plasmonic NPs. For instance, Ma et al. have concluded that the oxygen-deficient ZnO as a passivation layer can remarkably improve the surface water oxidation kinetics of the BiVO_4_/WO_3_ heterostructure.^17^ Zhang et al. have reported that the decoration of a cobalt phosphate (Co-Pi) cocatalyst on the conformally BiVO_4_ layered WO_3_ nanoplates promoted the water oxidation kinetics and photostability.^18^ In a study utilizing plasmonic NPs, the large Au NPs at the bottom layer served as a reflector and current collector, while the small Au NPs at the top layer functioned as an antenna, thereby, a strong electromagnetic field was induced due to the coupling interaction between the reflector and antenna, which facilitated the charge separation and enhanced the PEC activity of the BiVO_4_/WO_3_ thin film.^19^ Furthermore, decoration of Au NPs onto BiVO_4_ by the citrate method resulted in strong metal/support interaction and the hybrid nanocomposite generated a photocurrent of 6.25 mA cm^–2^ at 1.23 V vs RHE after FeOOH/NiOOH OER cocatalyst modification.^20^ The surface plasmon resonance (SPR) phenomenon is intrinsically related to the plasmonic NPs and is typically observed between 517 and 575 nm for Au NPs.^21^ In addition to the SPR advantage, a Schottky barrier naturally forms at the interface of the metal/semiconductor junction, increasing the charge separation rate and contributing to the photocurrent for water splitting. Although Au NPs-deposited WO_3_-based systems have demonstrated significant potential for PEC water splitting, conventional methods for incorporating plasmonic Au NPs such as electrodeposition,^22^ photodeposition,^23^ and citrate reduction^24^ suffer from limitations like low deposition rate or extreme experimental conditions. These challenges hinder precise control over the size, distribution, and uniformity of the Au NPs, which impacts overall PEC efficiency.

In this study, we introduce a novel and straightforward fabrication of the Au/BiVO_4_/WO_3_ ternary junction using an innovative electrochromic activation method for Au NPs integration. This approach operates under milder conditions and ensures a uniform distribution of Au NPs with a small average size (40 nm) on the BiVO_4_/WO_3_ heterostructure, mainly at the tips of the WO_3_ nanobricks. Furthermore, we systematically varied the number of consecutive drop-casting steps to optimize the amount of BiVO_4_ on fluorine-doped tin oxide (FTO) conductive glass and WO_3_, while maintaining a constant thickness for WO_3_. This optimization aimed to establish a correlation between the morphological and crystallinity features and the enhanced PEC water-splitting performance of the BiVO_4_/WO_3_ photoanode. Notably, the optimal photocurrent obtained from the BiVO_4_-2c/WO_3_ (BiVO_4_/WO_3_ heterostructure with 2 layers of BiVO_4_) configuration further increased upon decoration with plasmonic Au NPs. This study provides insights into the role of plasmonic metals in improving the efficiency of semiconductor-based heterojunctions and offers a novel approach for optimizing the design of advanced PEC systems.

## 2 Experimental Section

All chemicals were of analytical grade and used without further purification. Throughout all experiments, deionized water (pH 7.3, 18.3 MΩ cm^–1^) in the laboratory was sourced on a Milli-Q ultrapure (Type 1) water purification system (Biopak Polisher, Merck, USA). Before use, the FTO (surface resistivity ∼7 Ω sq^–1^, Sigma-Aldrich) coated glass substrates were ultrasonically cleaned with isopropyl alcohol, acetone, and a mixture of alkaline concentrate (Hellmanex III) and deionized water for 10 min each.

### 2.1 Synthesis of the WO_3_ Nanobricks Photoanode

The WO_3_ nanobricks were synthesized by a simple hydrothermal method using a modified version of a previously described procedure.^25^ In a typical procedure, 0.4 g of sodium tungsten (Na_2_WO_4_·2H_2_O, 95%, Alfa Aesar) and 0.2 g of oxalic acid (C_2_H_2_O_4_, 98%, Lachner), which acted as a stabilizer, were fully dissolved into 50 mL of deionized water with continuous stirring at room temperature. The growth solution was acidified to pH 2.5 by adding 2 mL of 3 M hydrochloric acid (HCl, 99%, mikroCHEM), resulting in the formation of a light-yellow precipitate almost 10 min later. The mixture was then poured into a 100 mL Teflon-lined stainless-steel autoclave, where a cleaned FTO substrate is obliquely positioned with its conductive side facing downward. The autoclave was tightly sealed and maintained at 180 °C for 12 h. The as-grown WO_3_ was rinsed with deionized water, dried in an oven at 60 °C, and then annealed in a muffle furnace at 500 °C for 1 h in an air atmosphere. The final WO_3_ nanobricks exhibited a light olive-green color.

### 2.2 Synthesis of the BiVO_4_/WO_3_ Heterojunction Photoanode

The BiVO_4_/WO_3_ heterostructures were fabricated by a facile drop-casting method as described elsewhere.^18^ In brief, 0.12 g of bismuth nitrate pentahydrate (BiNO_3_·5H_2_O, 98%, Sigma-Aldrich) and 0.08 g of vanadyl acetylacetonate ((VO(C_5_H_7_O_2_)_2_), 99%, Acros Organics) were dissolved in 5 mL of solution including acetic acid (CH_3_COOH, 99%, Penta) and acetylacetone (C_5_H_8_O_2_, 99%, Sigma-Aldrich) with a volume ratio of 20:1. During each deposition cycle, 10 μL of the solution mixture was dripped onto WO_3_, followed by annealing in a preheated furnace at 450 °C for 5 min and subsequent air cooling to room temperature. This process was consecutively repeated to achieve 1, 2, and 3 layers of BiVO_4_. After all coating cycles were completed, the films were further annealed in a muffle furnace at 550 °C for 2 h in air. The resulting heterostructures with 1, 2, and 3 layers of BiVO_4_ were designated as BiVO_4_-1c/WO_3_, BiVO_4_-2c/WO_3_, and BiVO_4_-3c/WO_3_, respectively. Additionally, the BiVO_4_ on FTO substrates with a varying number of layers (BiVO_4_-1c, BiVO_4_-2c, and BiVO_4_-3c) were also prepared with the same procedure.

### 2.3 Deposition of Plasmonic Au NPs on the BiVO_4_/WO_3_ Photoanode

**Scheme 1:**
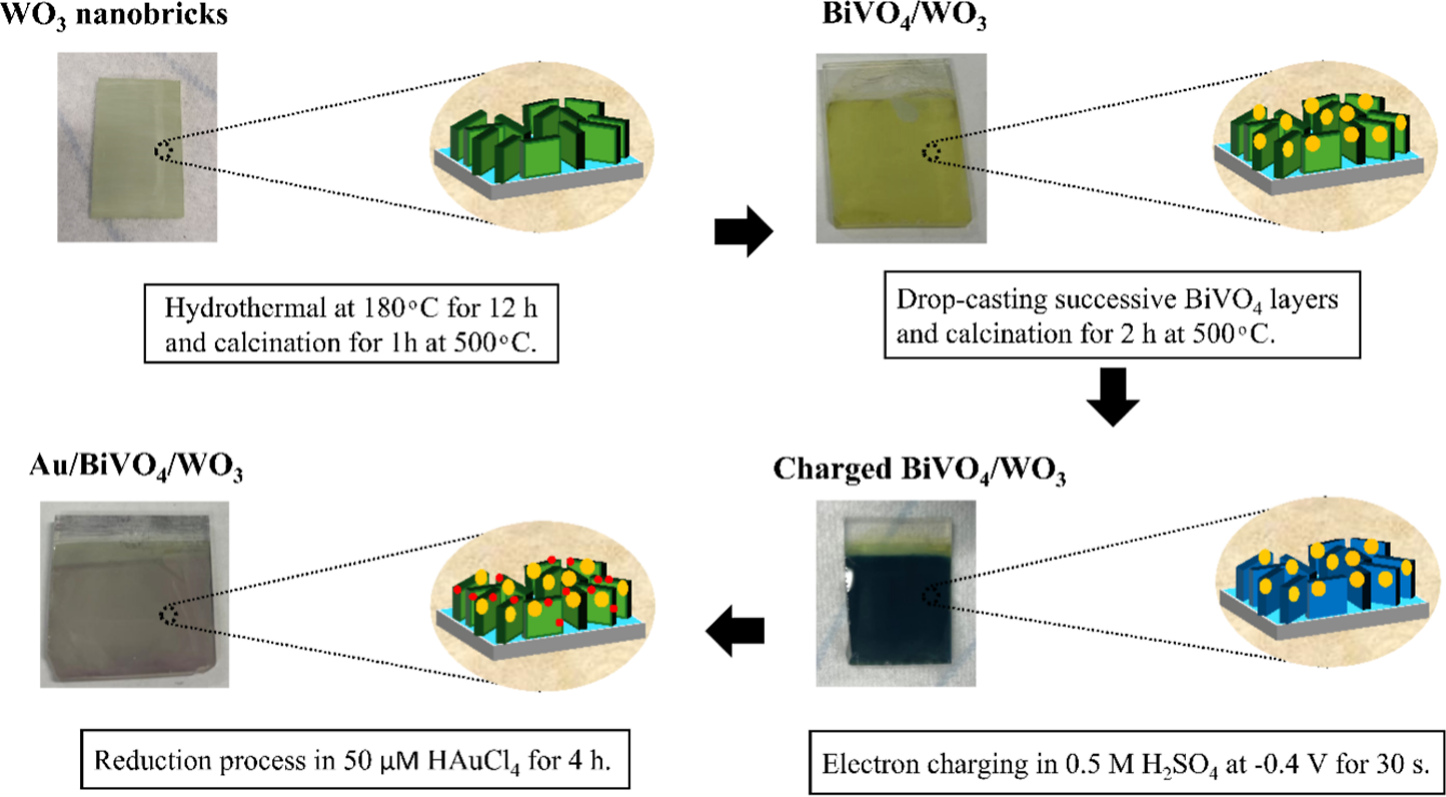
Schematic Representation for the Fabrication of the Au/BiVO_4_/WO_3_ Ternary Junction Photoanode

The Au NPs deposited BiVO_4_/WO_3_ heterostructure was fabricated by using a modified electron-charging method for WO_3_, which is based on cathodic electrochromism. Scheme 1 illustrates the synthesis process for constructing the Au/BiVO_4_/WO_3_ ternary junction. The electron-charging process was performed in chronoamperometric mode using a three-electrode setup in 0.5 M H_2_SO_4_ aqueous solution, with the BiVO_4_/WO_3_ acting as the working electrode, a platinum coil as the counter electrode, and an Ag/AgCl electrode (saturated with NaCl) serving as the reference. Figure S1a illustrates the cyclic voltammetry to determine the optimal charging potential of the BiVO_4_/WO_3_. Accordingly, the charging was carried out at –0.4 V vs Ag/AgCl for 30 s, as shown in Figure S1b. Following the charging process, the anode sample changed color from yellow to blue due to the intrinsic electrochromic property of WO_3_. The blue-colored BiVO_4_/WO_3_ was then immediately immersed into 50 μM of aqueous solution of gold chloride (HAuCl_4_·3H_2_O, Sigma-Aldrich) for 4 h in the dark. The uniform brown coloration of Au/BiVO_4_/WO_3_ indicated that electrons stored in WO_3_ were successfully released to form Au NPs upon reduction of the Au(III) precursor in solution (bleaching process). This electron-charging method essentially follows the literature protocol reported previously for WO_3_.^26^

### 2.4 PEC Performance Measurements

The PEC performance tests of BiVO_4_, WO_3_, BiVO_4_/WO_3_, and Au/BiVO_4_/WO_3_ photoanodes were conducted in a PEC cell (spectro-EC, Redox.me) by using a three-electrode configuration. In this setup, the samples served as working electrodes, Pt wire acted as the counter electrode, and Ag/AgCl (saturated with NaCl) was used as the reference electrode. Illumination was provided by a Pico solar simulator (G2 V Optics) with a standard AM 1.5G filter, delivering a light intensity of 87.5 mW cm^–2^. All measurements were performed on frontside illuminated electrodes with a constant area of ∼1.0 cm^2^. The aqueous solution of 0.5 M Na_2_SO_4_ at pH 7 was used as an electrolyte. The electrolyte was purged by nitrogen for 10 min prior to the measurements.

The potentials were converted to RHE using *V*_RHE_ = *V*_Ag/AgCl_ + 0.059 pH + 0.197, where *V*_RHE_ is the potential vs RHE, *V*_Ag/AgCl_ is the potential vs the Ag/AgCl electrode, and the pH is the pH of the electrolyte solution. The light-chopped linear sweep voltammograms (LSV) were recorded with a scan rate of 5 mV s^–1^ within the potential range of −0.5 to 1.0 V vs Ag/AgCl. The PEC performance of Au/BiVO_4_/WO_3_ was also examined under AM 1.5G conditions at 0.5, 1.0, and 1.5 sun, corresponding to 44.2, 87.5, and 129.1 mW cm^–2^, respectively.

The incident photon-to-current conversion efficiency (IPCE) tests were recorded under frontside illumination at 1.23 V vs RHE using a photoelectric spectrometer (Instytut Fotonowy Sp. z o.o.) equipped with a 150 W xenon lamp and a monochromator. The IPCE was measured using , where *J*_ph_ is the photocurrent density, *P*_mono_ is the monochromatic light intensity, and λ is the incident light wavelength. The applied bias photon-to-current efficiency (ABPE) was derived from the LSV curve and calculated using , where *J*_LSV_ is the photocurrent density under the applied potential of *V* and *P*_total_ is the total light intensity of the AM 1.5G solar spectrum. The photostability test of BiVO_4_/WO_3_ and Au/BiVO_4_/WO_3_ photoelectrodes was recorded by a chronoamperometric technique at 1.23 V vs RHE under continuous AM 1.5G light irradiation (87.5 mW cm^–2^) for 30 min.

Electrochemical impedance spectroscopy (EIS) data were scanned over a frequency range of 100 kHz to 0.1 Hz with an AC signal of 10 mV amplitude at 1.23 V vs RHE under illumination. Additionally, Mott–Schottky (MS) analysis was acquired in the dark using 20 potential steps at 1.5 kHz. The open-circuit potential (OCP) of the uncharged and charged BiVO_4_/WO_3_ electrodes was measured in the dark.

### 2.5 Characterization

The crystal structure of the metal oxides was analyzed using an X-ray diffractometer (XRD, Rigaku Miniflex 600) with a Co K_α_ irradiation source (λ = 0.17902 nm). The diffractometer operated at 40 kV and 100 mA, with a step size of 0.04°, and scans were conducted over a 2θ range from 20° to 80°. The microstructure of the nanocomposites was examined by high-resolution transmission electron microscopy (HRTEM, JEM-2100Plus, JEOL) and scanning electron microscopy (SEM, Nova NanoSEM 450) integrated with energy-dispersive X-ray spectroscopy (EDX). X-ray photoelectron spectroscopy (XPS, Axis Ultra DLD spectrometer, Kratos Analytical Ltd.), which was operated at 150 W (10 mA, 15 kV) and coupled with a monochromatized Al Kα radiation (*h*ν = 1,486.7 eV), was employed to analyze the elemental composition of the BiVO_4_/WO_3_ and Au/BiVO_4_/WO_3_ heterostructures. The binding energy was corrected with reference to the C 1s peak at 284.6 eV and the XPS spectra were processed by Casa XPS software. The surface potential mapping of nanostructures was obtained using Kelvin Probe Force Microscopy (KPFM, Dimension Icon from Bruker, Billerica, MA, USA), a noncontact atomic force microscopy technique. The work function of the CoCr tip was calibrated by the known work function of highly oriented pyrolytic graphite as a reference. The optical property measurements were conducted by a UV–vis–NIR spectrophotometer (Lambda 1050, PerkinElmer). The steady-state emission spectra were recorded using an RF-6000 Shimadzu spectrofluorophotometer at an excitation wavelength of 325 nm. The PEC and electrochemical measurements of the developed electrodes were evaluated by using an electrochemical workstation (SP-200 Potentiostat, BioLogic) equipped with EIS.

## 3 Results and Discussion

The ternary junction of Au/BiVO_4_/WO_3_ was integrated by a set of syntheses procedures where BiVO_4_ NPs were introduced on hydrothermally grown WO_3_ nanobricks, followed by the deposition of Au NPs through electrochromic activation of WO_3_. Depending on the amount of BiVO_4_ deposition, the number of BiVO_4_ drop-casting cycles was systematically varied from 1 to 3 on FTO and WO_3_ to achieve the best PEC water-splitting performance. As described in the experimental section, the BiVO_4_ and BiVO_4_/WO_3_ thin films with 1, 2, and 3 layers of BiVO_4_ were denoted as BiVO_4_-1c/WO_3_, BiVO_4_-2c/WO_3_, and BiVO_4_-3c/WO_3_, respectively. The PEC performance of Au/BiVO_4_/WO_3_ was compared with the best-performing photoanodes of BiVO_4_-2c and BiVO_4_-2c/WO_3_ in their irrespective series as provided in the Supporting Information (Figure S9a,b). Therefore, BiVO_4_-2c and BiVO_4_-2c/WO_3_ were used for the main characterization and referred to as BiVO_4_ and BiVO_4_/WO_3_ in the following sections, respectively.

The color of charged BiVO_4_/WO_3_ offers a qualitative representation of its charging state. A deeper blue color occurring at more negative potentials signifies a higher electron storage capacity, reflecting superior reducing capability of the electrochromic material. Figure S2a illustrates the color transition of BiVO_4_/WO_3_ thin films from yellow to blue at different charging potentials. Notably, the blueness of the BiVO_4_/WO_3_ heterostructure increases with increasingly negative applied potentials. In fact, the thermodynamic oxidizing or reducing ability of an electrode can be determined by its OCP, where a lower OCP corresponds to a greater reducing capacity.^26^Figure S2b demonstrates the OCP measurements of the charged BiVO_4_/WO_3_ samples under different charging potentials in the dark, revealing reasonably stable values over 30 s. Additionally, the dependence of the OCP on the applied charging potential is delineated in Figure S2c. As can be clearly noticed, the observed OCP value is decreasing with increasing charging potential and stabilizes around −0.6 V vs Ag/AgCl. Based on these results, the charging potential was selected as –0.4 V vs Ag/AgCl to integrate the Au NPs deposition under more moderate experimental conditions. Besides, it was observed that an applied potential more negative than −1 V leads to detachment of the sample from the substrate surface.

**Figure 1:**
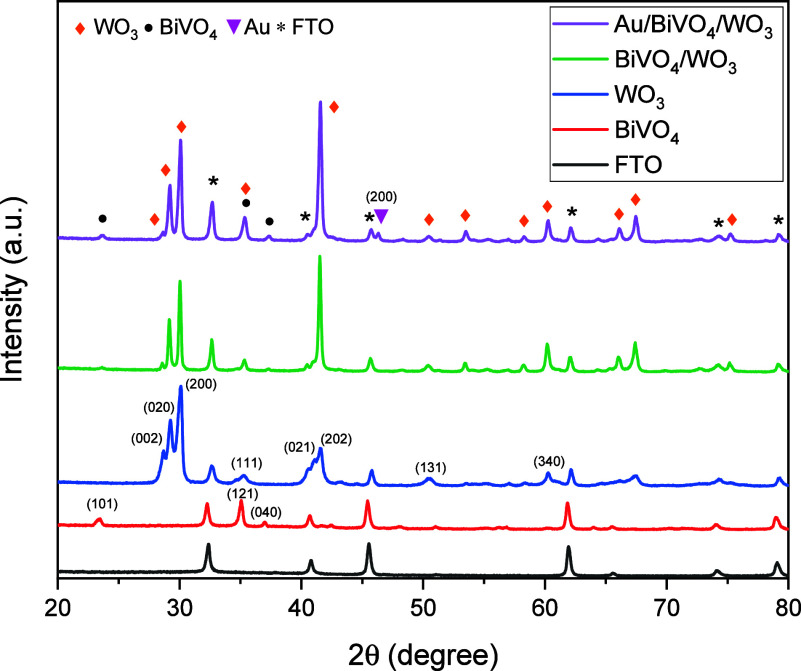
XRD patterns of WO_3_, BiVO_4_, BiVO_4_/WO_3_, and Au/BiVO_4_/WO_3_ photoanodes.

Figure 1 presents the XRD patterns of WO_3_, BiVO_4_, BiVO_4_/WO_3_, and Au/BiVO_4_/WO_3_. The diffraction peaks marked with an asterisk were assigned to the FTO substrate. The diffraction peaks at 28.6, 29.2, 30.2, and 35.2° correspond to the (002), (020), (200), and (111) plane of the monoclinic WO_3_ (JCPDS 72-0677), respectively. Besides, the peaks at 23.4°, 35.1°, and 37.1° correspond to the (101), (121), and (040) planes of the monoclinic BiVO_4_ (JCPDS 14-0688), respectively. These characteristic peaks of WO_3_ and BiVO_4_ are also evident in the XRD patterns of BiVO_4_/WO_3_ and Au/BiVO_4_/WO_3_. Additionally, the peak at 46.3° is indexed with the (200) plane of Au and is only observed in the Au/BiVO_4_/WO_3_ sample.^27^ The most dominant peak in the XRD patterns of WO_3_ is the (200) peak. The (202) peak of WO_3_ emerged as the most dominant peak for BiVO_4_/WO_3_ and Au/BiVO_4_/WO_3_. This represents the alignment in other crystallographic directions due to the second thermal treatment to crystallize BiVO_4_ onto WO_3_.

**Figure 2:**
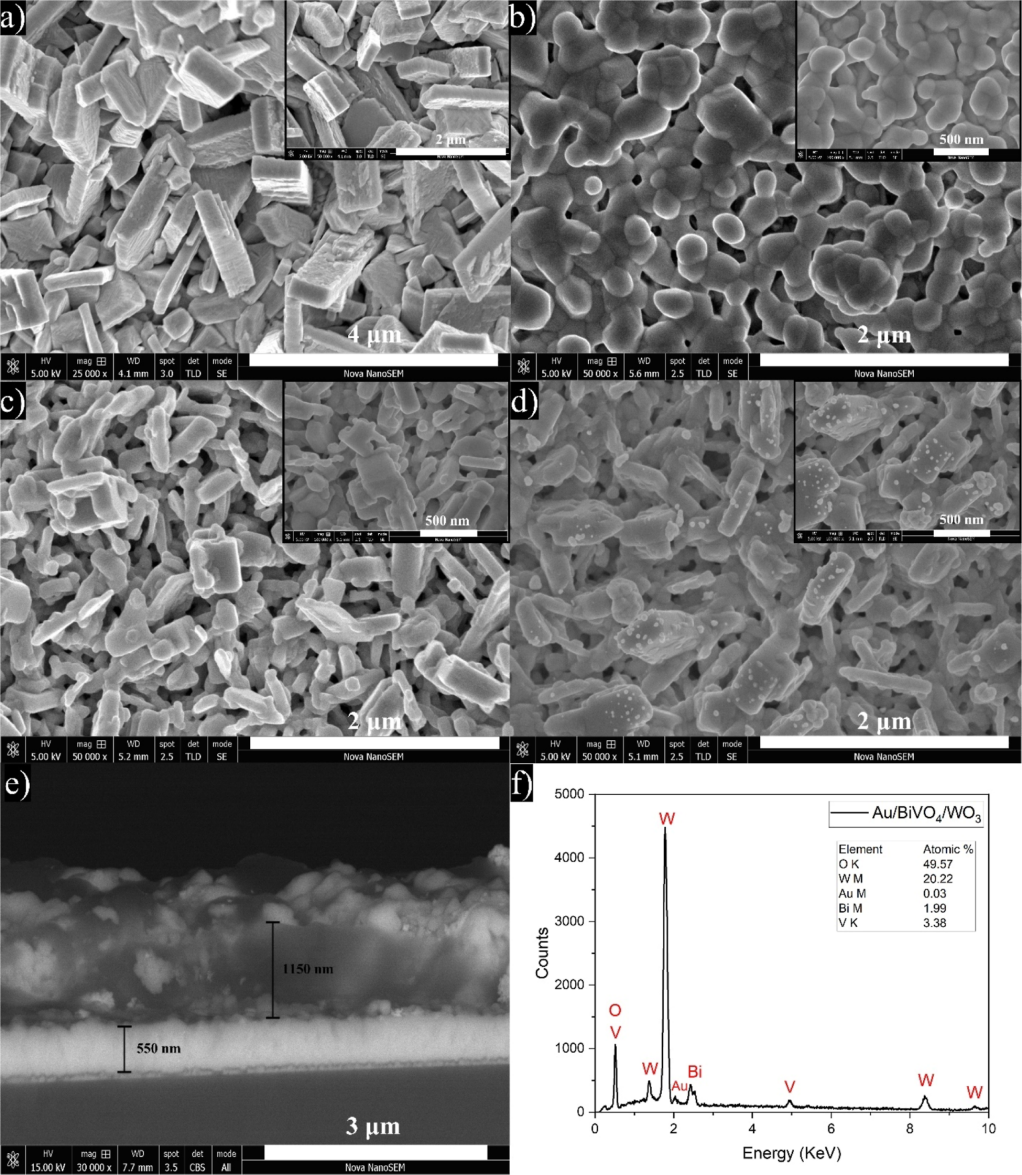
SEM image of (a) WO_3_, (b) BiVO_4_, (c) BiVO_4_/WO_3_, and (d) Au/BiVO_4_/WO_3_; (e) cross-sectional view and (f) EDX spectrum of Au/BiVO_4_/WO_3_. Insets are the high-magnification SEM images.

Figure S3a displays the SEM images of the as-grown WO_3_ on the FTO substrate. The as-grown WO_3_ is primarily composed of rectangular prism-shaped nanobricks, ranging from 1 to 2 μm in length (measured from the top side). These nanobricks are perpendicularly grown on the FTO substrate. After heat treatment, as can be seen in Figure 2a, the length of WO_3_ nanobricks was significantly reduced to 500–1000 nm, while the large voids between them were preserved. Figure S3b–d reveals that the amount of BiVO_4_ NPs on the WO_3_ nanobricks gradually increased with increasing coating cycles. Notably, these voids nearly disappeared for the BiVO_4_-3c/WO_3_ sample, leading to an aggregation of BiVO_4_ NPs and, thus, an obstruction of overall porosity. As shown in Figure 2b, BiVO_4_-2c has densely packed grains with an average size of 220 nm, closely adhering to the surface of the FTO substrate. On the other hand, Figure 2c illustrates that the surface of WO_3_ is uniformly covered by BiVO_4_ NPs with two successive cycles of drop casting. Interestingly, the Au/BiVO_4_/WO_3_ ternary junction photoanode exhibited no significant morphological change compared to BiVO_4_-2c/WO_3_, as displayed in Figure 2d. It is particularly noteworthy that almost circular Au NPs with an average size of 40 nm formed merely on the WO_3_ bricks when they were already decorated with BiVO_4_ NPs. This indicates that Au NPs were locally reduced by the electrons stored in WO_3_ during the bleaching process. A cross-sectional SEM image of the Au/BiVO_4_/WO_3_ in Figure 2e reveals that the thickness of Au/BiVO_4_/WO_3_ nanostructured film was 1150 nm over 550 nm of the FTO conductive layer. Figure S4 shows the SEM image of Au/BiVO_4_/WO_3_ in the backscattered electron mode. The mass contrast sensitivity in the backscatter mode is higher than in the secondary emission so that Au NPs appear shinier than the surrounding BiVO_4_ and WO_3_ textures. In addition, the EDX analysis of the Au/BiVO_4_/WO_3_ photoanode is presented in Figure 2f and confirms that the heterostructure is only composed of gold, bismuth, vanadium, tungsten, and oxygen with atomic ratios of 0.03, 1.99, 3.38, 20.22, and 49.57, respectively. In addition, Figure S5a displays the SEM image of Au/BiVO_4_/WO_3_ to estimate the elemental composition and the distribution of component elements. As can be seen, Au NPs are evenly scattered over the BiVO_4_/WO_3_ binary junction, dominantly over the head of the WO_3_ nanobricks. The presence and spatial distribution of O, W, Bi, V, and Au elements in the Au/BiVO_4_/WO_3_ heterostructure is also validated by EDX mapping results, as presented in Figure S5b–f. These findings corroborate the XPS results, which are discussed in the following section.

**Figure 3:**
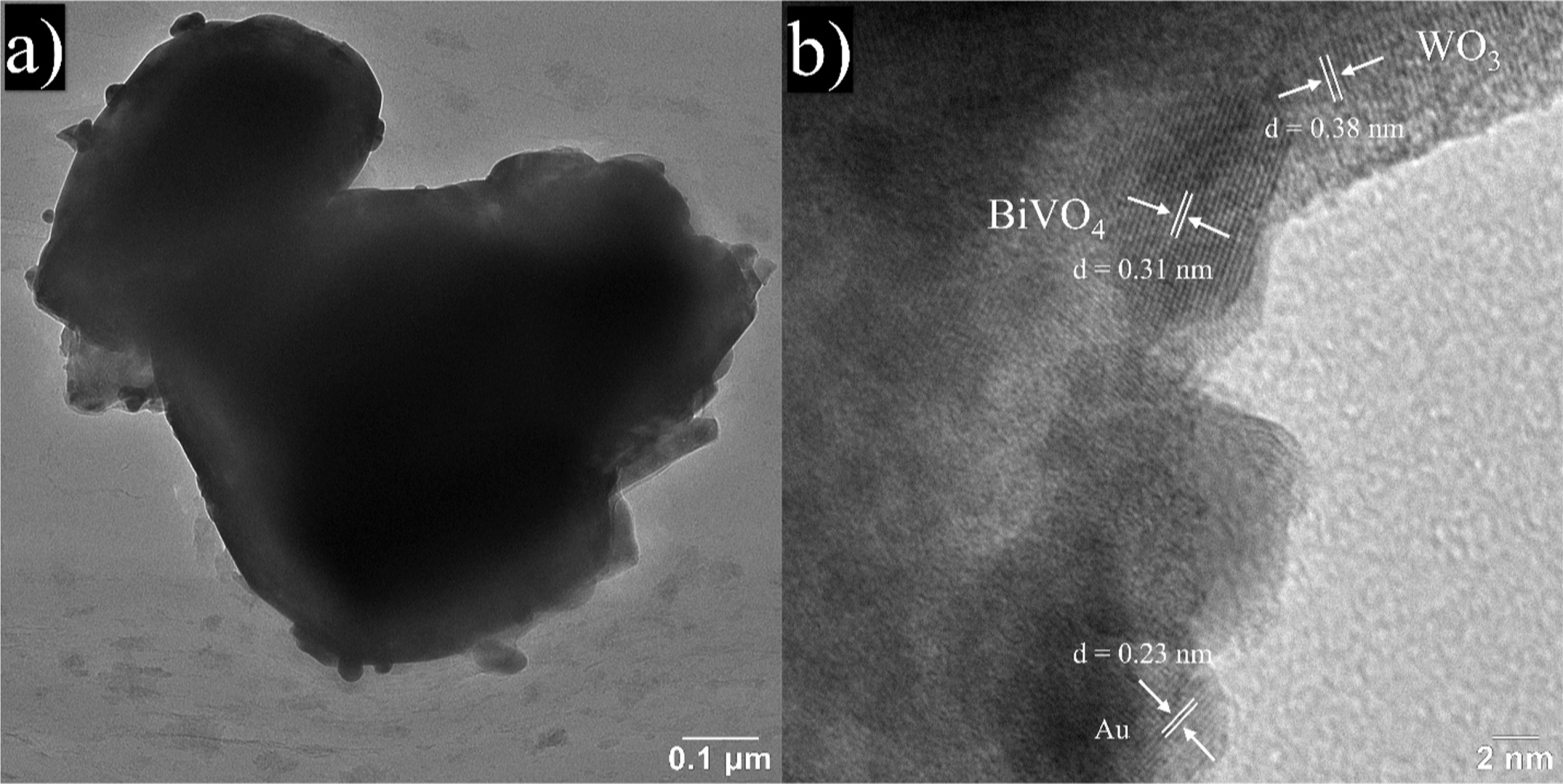
(a) TEM and (b) HRTEM images of Au/BiVO_4_/WO_3_.

To gain a more comprehensive view of the microstructure, the Au/BiVO_4_/WO_3_ nanoarchitecture photoanode was further investigated by using TEM and HRTEM. In Figure 3a, the TEM image illustrates the distinct nanobrick structure of the WO_3_ (dark contrast). Another clear observation is that BiVO_4_ NPs (bright contrast) and spherical Au NPs with small particle sizes are firmly attached to the edges of the nanobricks, corroborating the SEM findings. Additionally, in Figure 3b, the HRTEM image shows the measured *d* spacing values of ∼0.38, 0.31, and 0.23 nm, corresponding to the (002) plane of the WO_3_,^17^ (121) plane of the BiVO_4_,^28^ and (200) plane of the Au,^29^ respectively.

**Figure 4:**
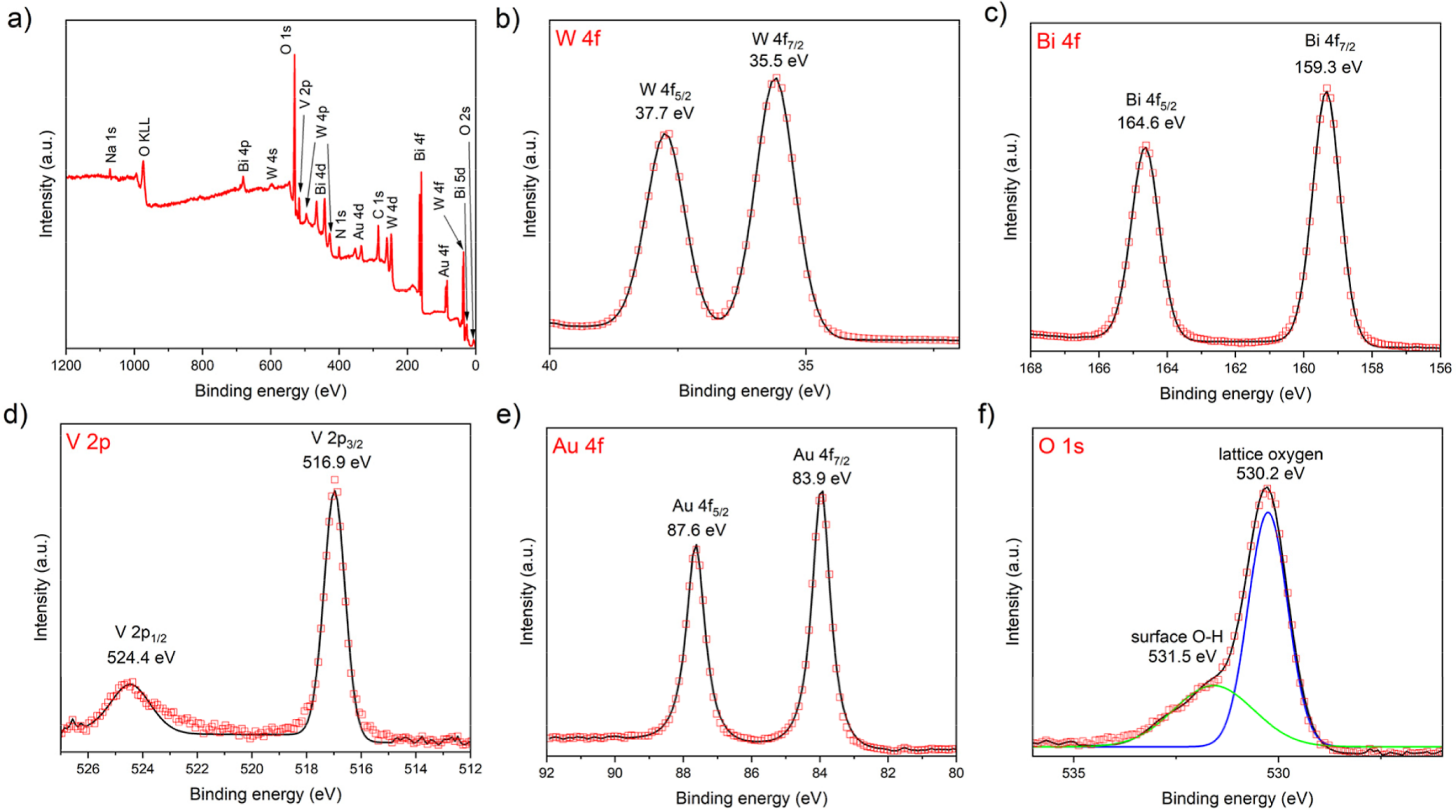
(a) XPS survey spectrum of Au/BiVO_4_/WO_3_, and high-resolution XPS spectra of (b) W 4f, (c) Bi 4f, (d) V 2p, (e) Au 4f, and (f) O 1s regions.

Figure 4a illustrates the XPS survey spectrum of the multilayered Au/BiVO_4_/WO_3_ photoanode. The spectrum displays distinct peaks of the constituent elements of W, Bi, V, Au and O, together with minor peaks for Na and N, which could originate from the glass substrate. In Figure 4b, the high-resolution XPS spectrum of W 4f provides broad doublet peaks at 37.7 eV (W 4f_5/2_) and 35.5 eV (W 4f_7/2_), corresponding to the W^6+^ oxidation state in WO_3_. Figure 4c,d depicts the Bi 4f and V 2p high-resolution spectra, respectively. The prominent peaks at 164.6 (Bi 4f_5/2_) and 159.3 eV (Bi 4f_7/2_) signify the Bi^3+^ oxidation state, while characteristic peaks at 524.4 (V 2p_1/2_) and 516.9 eV (V 2p_3/2_) confirm the V^5+^ oxidation state within BiVO_4_.^30^ In Figure 4e, the high-resolution XPS spectrum of Au 4f indicates the two major peaks at 87.6 and 83.9 eV, corresponding to the metallic state of Au.^31^ Besides, Figure 4f demonstrates the high-resolution XPS spectrum of O 1s, where the deconvoluted peaks at 531.5 and 530.2 eV are attributed to lattice oxygen and surface hydroxyl (−OH) groups.^32^ Comparatively, the BiVO_4_/WO_3_ photoanode was also analyzed by XPS, and the results are shown in Figure S6a–e. No additional impurity was detected, and no significant difference in peak positions was observed. Notably, the core level binding energies are influenced by the band bending, and the changes of binding energies upon deposition of Au can be therefore used for tracking possible changes in the band bending.^33,34^ The comparison of XPS binding energies of elements in BiVO_4_/WO_3_ and Au/BiVO_4_/WO_3_ is presented in Table S1. It was observed that the inclusion of Au NPs did not change significantly (within the XPS experimental error of ∼0.2 eV) the core level binding energies of BiVO_4_/WO_3_, suggesting that the deposition of Au NPs did not induce any significant changes of the band bending.

**Figure 5:**
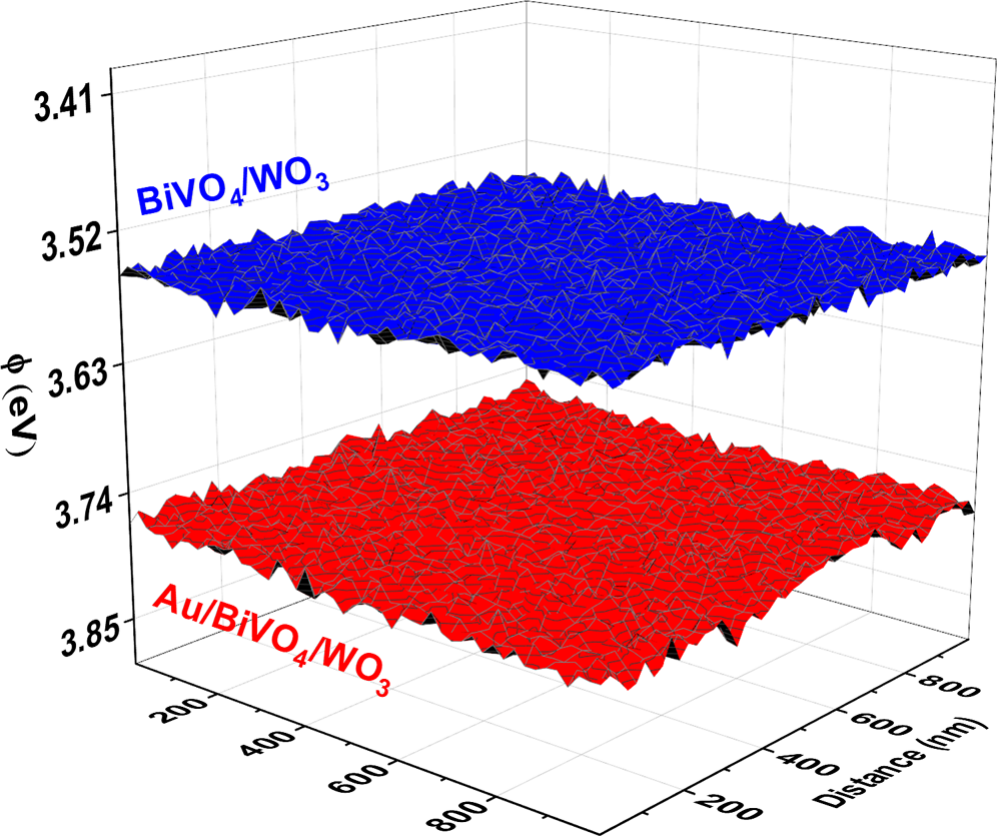
Work function with respect to variations in CPD over BiVO_4_-2c/WO_3_ and Au/BiVO_4_/WO_3_ heterostructures.

However, the changes in surface energetics upon the deposition of Au NPs can be followed more precisely by measuring the changes in the work function using KPFM which typically has a much higher energy resolution (∼0.002 eV) with respect to XPS (∼0.2 eV). Figure 5 illustrates the work function mapping conducted over a 1 μm^2^ area of the photoanode surface. The color variations across the surfaces of the fabricated binary and ternary junctions indicate fluctuations in their work function. The measured work function values for BiVO_4_/WO_3_ and Au/BiVO_4_/WO_3_ are 3.56 and 3.77 eV, respectively. The introduction of Au NPs onto the BiVO_4_/WO_3_ leads to a work function increase of 0.21 eV. This means that the local deposition of Au NPs on WO_3_ nanobricks due to the electrochromism effect either leads to the formation of an additional negative surface dipole, or—with the same effect—to the formation of a Schottky junction between WO_3_ nanobricks and Au NPs, or to a combination of these two processes. A Schottky barrier typically arises at the semiconductor–metal interface when the work function of the metal is larger than the work function of the semiconductor. Indeed, the work functions of WO_3_ nanoplates and Au NPs were reported to be 4.81 eV for 5.13 eV, respectively.^31^ We therefore assume that the formation of a Schottky junction with a relatively small barrier (∼0.21 eV) is highly likely. The resulting electric field can further promote the spatial separation of charge carriers within the Au/BiVO_4_/WO_3_ by modulating the gradients of *quasi*-Fermi levels of electrons and holes and driving the photogenerated holes to the interface and the photogenerated electrons toward the bulk of BiVO_4_/WO_3_.

Figure S7a provides the UV–vis diffuse reflectance spectra of the BiVO_4_ photoanode series. The absorbance increases as the number of BiVO_4_ layers increases. The thickness of these films was evaluated from their cross-sectional SEM images. The absorbance at a specific wavelength (*A*_λ_) is related to the absorption coefficient (α) and the thickness (*d*) by the equation of *A*_λ_ = α_λ_ × *d*. Using this linear relationship, the absorption coefficient of BiVO_4_ at 420 nm (α_420_ = 5.25 × 10^3^ cm^–1^) was calculated from the slope of the absorbance–thickness plot in Figure S8. The α_420_ value is in good agreement with the previously reported results.^14^ Besides, the absorbance of BiVO_4_/WO_3_ at 420 nm is slightly increasing with the gradually increasing BiVO_4_ layers, as can be seen from Figure S7b. It is essential to realize that WO_3_ also effectively contributes to the overall absorption of the BiVO_4_/WO_3_ photoanode at 420 nm. Therefore, to estimate the loading amount of BiVO_4_ layers (based on their individual thickness) on WO_3_, this contribution was subtracted from the total absorption. The obtained values are provided in Table S2, proving that the loading amount of BiVO_4_ NPs onto WO_3_ nanobricks progressively increases with respect to the increase in the number of BiVO_4_ drop-casting cycles. In fact, the calculated thickness corresponds to the rough thickness of the heterostructures.

**Figure 6:**
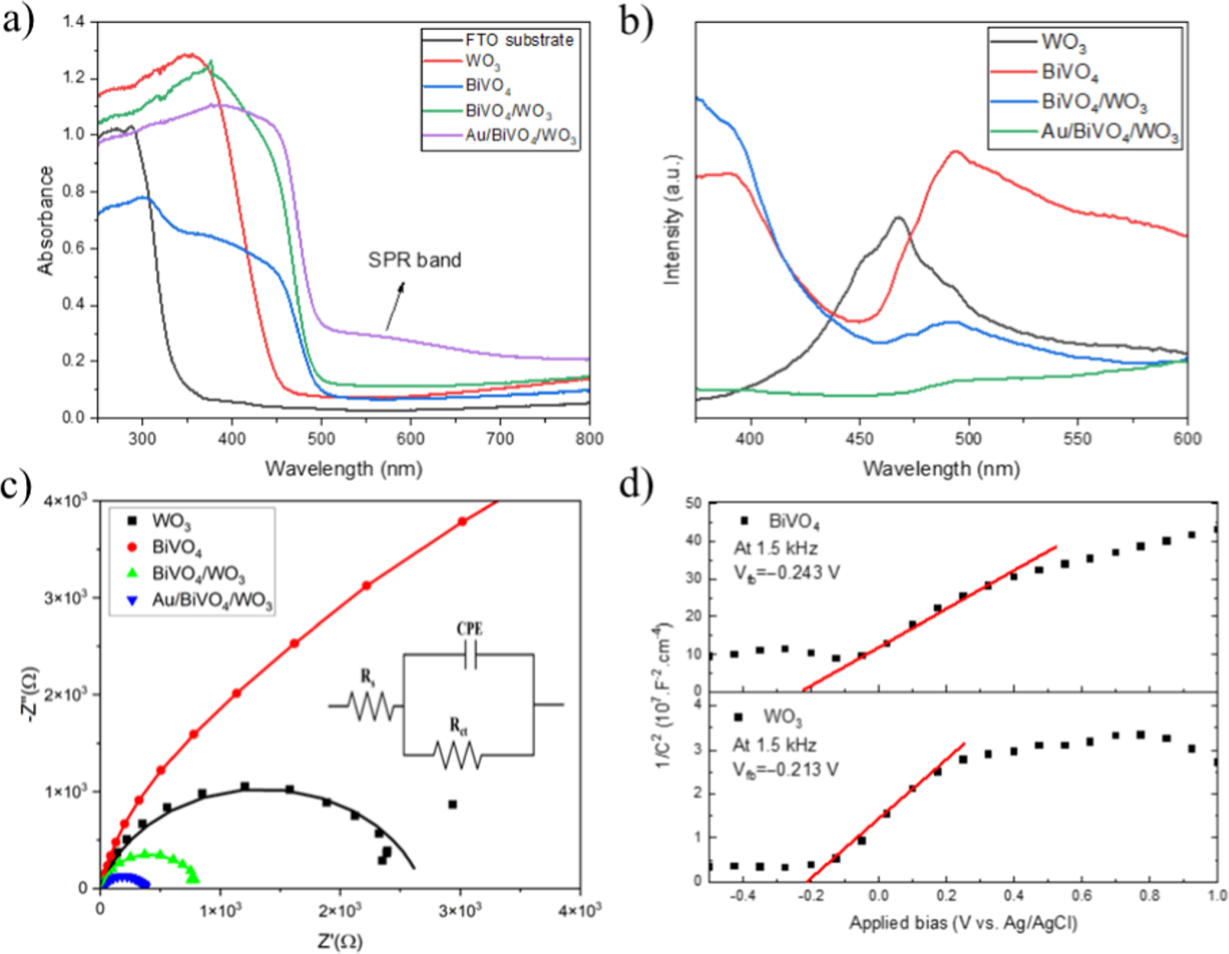
(a) Absorbance spectra (with the FTO substrate), (b) PL spectra (at an excitation wavelength of 325 nm), and (c) EIS curves in the form of Nyquist plots (points) with model fits (lines) under AM 1.5G illumination at 1.23 V vs RHE for all the samples; (d) MS analyses of the pristine BiVO_4_ and WO_3_.

The UV–vis diffuse reflectance spectra of the BiVO_4_, WO_3_, BiVO_4_/WO_3_, and Au/BiVO_4_/WO_3_ photoanodes and the FTO substrate are shown in Figure 6a. It is observed that the FTO thin film with a thickness of 550 nm has no significant light absorption in the spectral range of 400–800 nm where it could potentially overlap with the absorption of the produced visible light active materials. The absorption band edge of WO_3_ appeared at 450 nm, corresponding to its band gap of 2.75 eV. The absorption band edge of BiVO_4_ appeared at 500 nm, corresponding to its band gap of 2.48 eV. The BiVO_4_/WO_3_ heterojunction exhibited a considerably enhanced light absorption capability compared to pristine forms of WO_3_ and BiVO_4_. Typically, the broad peak at 570 nm in the absorption spectrum of Au/BiVO_4_/WO_3_ was attributed to the SPR band of the Au NPs whose size is around 40 nm. These findings suggest that the incorporation of Au NPs can extend the light absorption range of BiVO_4_/WO_3_, promoting its ability to utilize a larger portion of the solar spectrum.

To explore the charge separation behavior within the WO_3_, BiVO_4_, BiVO_4_/WO_3_, and Au/BiVO_4_/WO_3_ nanostructures, PL measurements were collected under the excitation wavelength of 325 nm and shown in Figure 6b. There is only one wide and intense emission peak appearing at 460 nm in the spectrum of WO_3_. This peak was ascribed to the near band edge (NBE) transition of WO_3_.^35^ Similarly, two broad emission peaks appeared in the spectrum of BiVO_4_. The peaks at 400 and 495 nm are associated with the deep level defects and NBE of the BiVO_4_, respectively.^36^ In fact, the lower PL intensity refers to the higher separation and lower recombination rates of the photogenerated electron–hole pairs. The PL intensities of BiVO_4_/WO_3_ around the distinctive NBE peaks are less pronounced compared to those of the pristine WO_3_ and BiVO_4_, suggesting an effectively diminished charge recombination due to the heterojunction formation between these two metal oxides. Moreover, the PL intensity of all observed emission peaks was further decreased in the spectrum of Au/BiVO_4_/WO_3_, accelerating the charge separation, as discussed in Figure 5.

Figure 6c presents the EIS curves in the form of Nyquist plots (points) along with their model fits for the produced photoelectrodes under AM 1.5G illumination at 1.23 V vs RHE. The semicircular arcs were fitted to a simplified equivalent circuit model (inset of Figure 6c). Herein, *R*_s_ represents the resistance of the electrolyte solution, constant phase element represents the double layer behavior for the inhomogeneous electrode surface, and *R*_ct_ in our case represents the combined charge transport and interfacial charge transfer resistance. The observed *R*_ct_ values were about 19959 Ω, 2690 Ω, 786 Ω, and 357 Ω for BiVO_4_, WO_3_, BiVO_4_/WO_3_, and Au/BiVO_4_/WO_3_, respectively. The BiVO_4_/WO_3_ heterojunction demonstrated a significantly lower *R*_ct_ value compared to BiVO_4_ and WO_3_, which suffer from poor charge kinetics. The *R*_c_ value was further diminished upon inclusion of Au NPs on BiVO_4_/WO_3_, implying the most rapid charge transfer. It is inferred that the high conductivity of Au NPs could improve the electrical properties and enhance the charge extraction at the interface between the photoelectrode and electrolyte.

The MS measurements of BiVO_4_ and WO_3_ films were performed in the dark at 1.5 kHz, assuming that the MS formalism for the determination of the flat-band potential can be applied to our electrodes consisting of rather large and compactly annealed particles (Figure 2). As shown in Figure 6d, both pristine materials demonstrated an n-type semiconducting nature with positive slopes in their MS plots. Based on the MS equation,^37^ the intercept of this plot on the *x* axis represents the flat-band potential (*V*_fb_), and its slope represents the donor density (*N*_D_). The estimated *V*_fb_ values for BiVO_4_ and WO_3_ were −0.243 and −0.213 V vs Ag/AgCl, respectively. Besides, the estimated *N*_D_ values for BiVO_4_ and WO_3_ were 3.26 × 10^19^ cm^–3^ and 4.45 × 10^19^ cm^–3^, respectively. In n-type semiconductors, the energy difference between the conduction band (CB) edge and *V*_fb_ is typically very small (∼0.1 eV).^38,39^ Consequently, the CB edge of BiVO_4_ is slightly more negative than that of WO_3_, which is in line with previously published results.^40,41^ The heterostructure between BiVO_4_ and WO_3_ should therefore enable an effective extraction of the photogenerated electrons from the CB of BiVO_4_ to the CB of WO_3_, resulting in an increased PEC performance of BiVO_4_/WO_3_. It should be emphasized that MS formalism is proposed for a planar geometry of the semiconductor and electrolyte junction. However, a vast amount of surface states or highly porous nanostructure can lead to nonlinearities in the MS plot.^37^ Indeed, WO_3_ nanobricks with lengths of several hundreds of nm are large enough to resemble the compact electrode morphology (Figure 2a).

The band edge positions determine the feasibility of charge transfer between the constituent semiconductors within the heterostructure; hence, the CB minima (*E*_CBM_) and the VB maxima (*E*_VBM_) of WO_3_ and BiVO_4_ were estimated using their flat-band potentials obtained from MS plots. As we also stated above, the flat-band potential of the n-type semiconductor is anticipated to lie ca. 0.1 V below the CB edge.^42,43^ Accordingly, the *E*_CBM_ of WO_3_ and BiVO_4_ were estimated to be 0.29 V vs RHE and 0.26 V vs RHE at pH 7, respectively. Subsequently, the VB edge was estimated by using the observed band gap values and CB edges. Therefore, the *E*_VBM_ of WO_3_ and BiVO_4_ were found to be 3.04 V vs RHE and 2.74 V vs RHE at pH 7, respectively. These values are consistent with the earlier reported values,^44^ implying that the BiVO_4_/WO_3_ nanostructure is characterized by a typical type-II band alignment.

**Figure 7:**
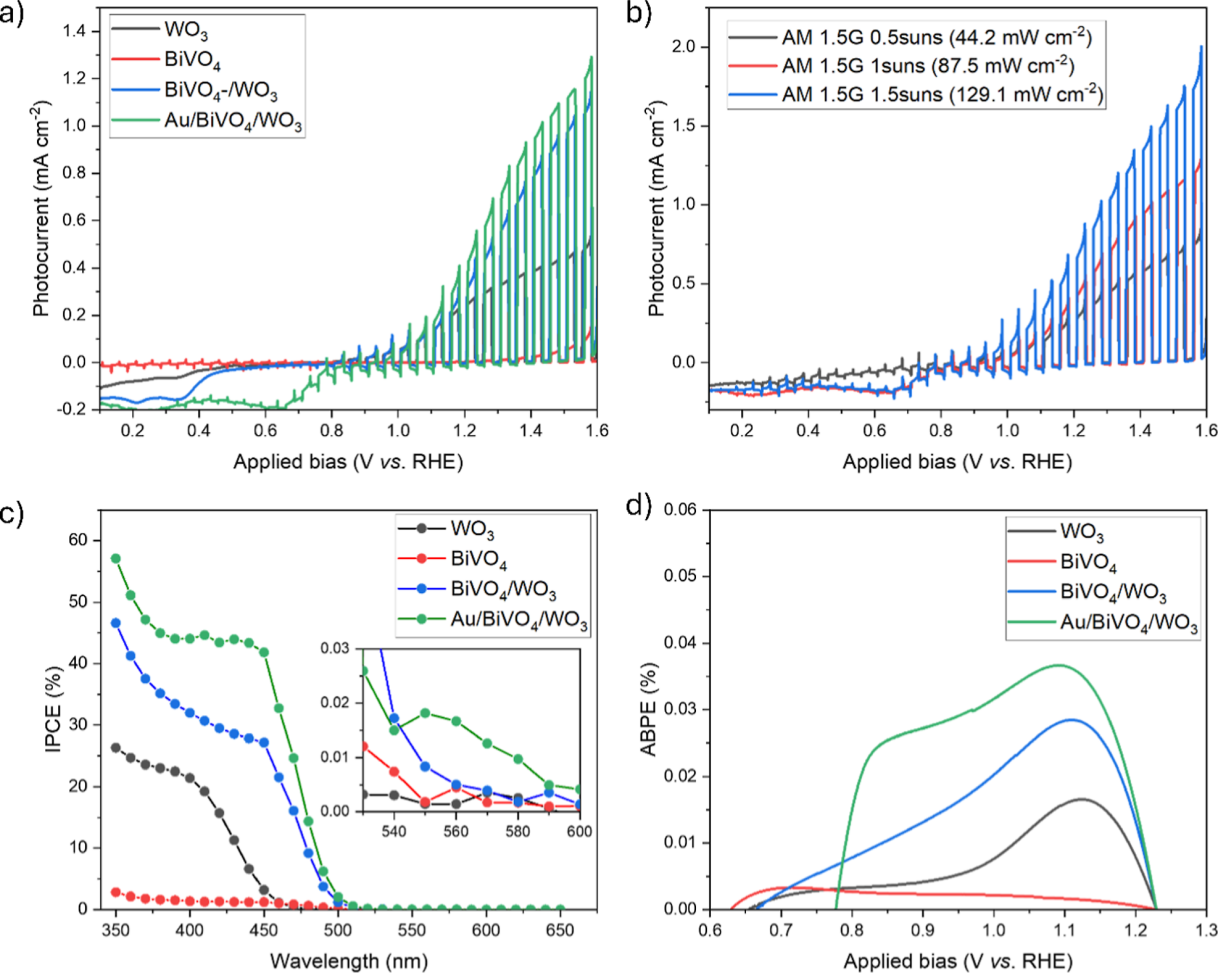
(a) Light-chopped LSV curves of the developed photoanode thin films under AM 1.5G illumination, (b) light intensity dependence of the photocurrent obtained from the Au/BiVO_4_/WO_3_ ternary junction, (c) incident photon to current conversion efficiency (IPCE) plots recorded at 1.23 V vs RHE, and (d) ABPE plots. All PEC measurements were carried out in a 0.5 M Na_2_SO_4_ aqueous solution (pH 7).

The PEC water-splitting performance of the optimized photoanodes was evaluated by the light-chopped LSV curves under AM 1.5G irradiation (1 sun), as provided in Figure 7a. The photocurrent response of all photoanodes increases with an increasing applied bias. No dark current was observed in the light-off cycles, signifying that the obtained currents originate only from the charge carriers generated upon illumination. The pristine BiVO_4_ possessed the lowest photocurrent density of 0.008 mA cm^–2^ at 1.23 V vs RHE. This photoactivity was assigned to the granular structure of BiVO_4_ with an average particle size of 220 nm (Figure 2b) that is greater than the hole diffusion length (∼100 nm) in BiVO_4_,^45^ limiting its photocurrent due to the poor hole transport. Fabricating a heterojunction of BiVO_4_/WO_3_ significantly improved the photocurrent compared to its pristine constituents. BiVO_4_/WO_3_ exhibited a photocurrent density of 0.438 mA cm^–2^ at 1.23 V vs. RHE, which is nearly 1.5 times higher than that of WO_3_ (0.295 mA cm^–2^) at 1.23 V vs RHE. The incorporation of Au NPs on BiVO_4_/WO_3_, particularly reduced on WO_3_, is evinced to be a useful strategy since Au/BiVO_4_/WO_3_ delivered the highest photocurrent of 0.578 mA cm^–2^ among the four electrodes. The enhanced PEC performance obtained from the ternary junction was mainly associated with the broad light harvesting capacity, efficient charge separation, and rapid interfacial charge kinetics. The comparison of the PEC water-splitting efficiency of the Au/BiVO_4_/WO_3_ photoanode with the advanced WO_3_-based photoanodes previously reported in the literature is presented in Table S3.

The light intensity-dependent photocurrent efficiency of the best-performing Au/BiVO_4_/WO_3_ sample is shown in Figure 7b. The light-chopped LSV curves were collected under AM 1.5G irradiation at 0.5, 1.0, and 1.5 sun conditions corresponding to light intensities of 44.2, 87.5, and 129.1 mW cm^–2^, respectively. The photocurrent is indeed found to increase linearly as a function of the intensity, reaching up to ∼ 0.71 mA cm^–2^ at 1.5 sun. This suggests that no detrimental effects of high intensity irradiation (*e.g*., enhanced recombination) are operative in this light intensity range.

To gain insight into the spectral photoresponse of the four electrodes, IPCE tests were carried out at 1.23 V vs RHE in a 0.5 M Na_2_SO_4_ aqueous solution (pH 7), and the results are illustrated in Figure 7c. It is deduced that the onset wavelength of WO_3_ appeared at ∼460 nm, while the other three photoanodes displayed an extended onset wavelength of ∼505 nm, in line with their absorption edge (Figure 6a). The IPCE value of the photoanodes at 400 nm for Au/BiVO_4_/WO_3_, BiVO_4_/WO_3_, WO_3_, and BiVO_4_ is ∼44.0, 32.7, 22.3, and 1.8%, respectively. Moreover, the SPR-related IPCE slight enhancement for Au/BiVO_4_/WO_3_ became apparent in the enlarged view of the IPCE curves (inset of Figure 7c) in the range of 540–600 nm, implying that the hot electrons of Au NPs can be effectively injected into the BiVO_4_/WO_3._

Figure 7d illustrates the comparison of ABPE performance of the synthesized photoanodes under irradiation, which represents from the practical point of view the most important performance metrics for water-splitting photoanodes. The maximum ABPE values for Au/BiVO_4_/WO_3_, BiVO_4_/WO_3_, WO_3_, and BiVO_4_ are 0.036% (at 1.09 V vs RHE), 0.028% (at 1.11 V vs RHE), 0.016% (at 1.12 V vs RHE), and 0.003% (at 0.70 V vs RHE), respectively. In other words, the best-performing Au/BiVO_4_/WO_3_ photoanodes exhibited an enhancement by a factor of 12 and 2.3 with respect to pristine BiVO_4_ and WO_3_ photoanodes, respectively.

A photostability test is important for assessing the prospects for the long-term durability of a photoelectrode against photocorrosion. Figure S10 presents the photostability curves of BiVO_4_/WO_3_ and Au/BiVO_4_/WO_3_ heterostructure photoanodes. Both electrodes maintained their initial photocurrent densities under a continuous AM 1.5G illumination for at least 30 min at 1.23 V vs RHE, indicating their good resilience to degradation.

**Figure 8:**
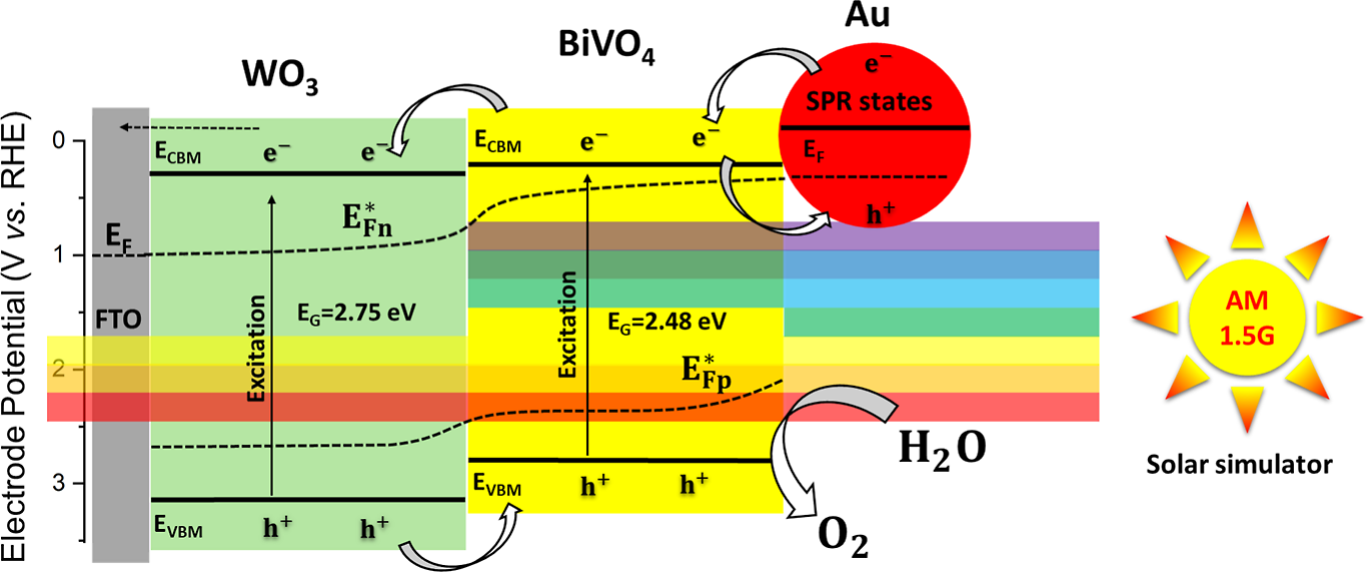
Schematic depiction of energy band diagram and charge transfer processes within the Au/BiVO_4_/WO_3_ ternary junction photoanode under frontside AM 1.5G illumination at 1.0 V vs RHE. The *E*_CBM_ positions of WO_3_ and BiVO_4_ were estimated from the flat-band potential measurements (Figure 6d) assuming that the *E*_CBM_ is close (ca. 0.1 V) to the *V*_fb_. Note that any band bending effects are omitted for the sake of clarity; the fundamental driving force for the charge separation is the gradient of the *quasi*-Fermi levels of electrons (*E_Fn_, i.e., the electrochemical potential of electrons) and holes (*E_Fp_, i.e., the negative of the electrochemical potential of holes).^46^

The energy band diagram and charge transfer process within the Au/BiVO_4_/WO_3_ ternary junction is proposed and illustrated in Figure 8. The VB and CB positions were calculated based on the flat-band and optical measurements discussed above. The estimated type-II band alignment is consistent with the previously proposed heterojunction formation between BiVO_4_ and WO_3_,^47,48^ resulting in a viable charge transfer pathway for an enhanced PEC water-splitting performance. The electron–hole pairs are generated in the BiVO_4_ and WO_3_ layers upon AM 1.5G irradiation. The photogenerated electrons from the CB of BiVO_4_ flow toward the CB of the pristine WO_3_ and are directed to the external circuit throughout FTO conductive glass, driving the HER. Simultaneously, the photogenerated holes in the VB of WO_3_ migrate to the VB of BiVO_4_, driving the OER. In addition, the incorporation of Au NPs can produce hot electrons under visible light through the SPR process to increase the light absorption range of the heterostructure, which was depicted by the attenuation of the green color. The photogenerated hot electrons in the SPR state of Au NPs are expected to nourish the charge carrier density in the optimized BiVO_4_/WO_3_ electrode, increasing the overall PEC water-splitting efficiency. Moreover, the gradient of the *quasi*-Fermi level of electrons drives the electrons toward the positively biased FTO. This alignment also allows the photogenerated electrons to easily flow from the SPR states of Au NPs (and subsequently BiVO_4_ and WO_3_) into the FTO substrate, minimizing recombination losses and promoting efficient charge extraction. At the same time, the photogenerated holes are driven by the gradient of the *quasi*-Fermi level of holes toward the semiconductor/electrolyte interface and induce water oxidation to oxygen.^46^

The Au/BiVO_4_/WO_3_ ternary junction introduced in our study is unique in its combination of components, fabrication methodology, and performance. Our innovative use of electrochromic activation to integrate Au NPs, a rarely investigated but highly effective method, resulted in synergistic improvements in light absorption, charge separation, and interfacial charge kinetics. This distinguishes our work from previous studies, in which Au NPs were deposited on WO_3_-based systems by conventional methods such as electrodeposition, photodeposition, and citrate reduction. These conventional methods often suffer from limitations, such as low deposition rate or harsh experimental conditions, which makes the control over the size, distribution, and uniformity of the Au NPs very challenging. The electrochromic deposition method described in this work is carried out under mild experimental conditions without any assistance from the reducing agent and facilitates a uniform distribution of the tiny Au NPs over WO_3_. Moreover, the size and density of the Au NPs can be controlled by adjusting the charging parameters or the concentration of the aqueous gold salt solution.^26^ We demonstrated that the Au/BiVO_4_/WO_3_ nanostructure achieved a substantial enhancement in photocurrent density, underscoring the scalability and practicality of this method for PEC water splitting.

## 4 Conclusions

In summary, the development of the Au/BiVO_4_/WO_3_ ternary junction demonstrated significant advancements in the PEC performance through a series of straightforward fabrication processes. BiVO_4_ NPs were applied to hydrothermally grown WO_3_ nanobricks via a drop-casting method, followed by the reduction of Au NPs onto the BiVO_4_/WO_3_ heterostructure through electrochromic activation of WO_3_. The systematic optimization of BiVO_4_ deposition on FTO conductive glass and WO_3_ further enhanced the photoanode’s performance, underscoring the critical role of morphological and optical features. The superior photocurrent achieved with this novel nanocomposite was attributed to several factors: (1) strong light absorption, owing to the BiVO_4_ photosensitizer and the SPR effect of Au NPs; (2) reduced charge recombination, leading to more efficient charge separation due to the Schottky barrier at the metal/semiconductor interface; and (3) rapid interfacial charge kinetics resulting from the high conductivity of WO_3_. Thus, this study provides insights into the role of plasmonic metals in enhancing the efficiency of semiconductor-based heterojunctions and offers a novel, scalable approach for optimizing the advanced PEC system design.

## Data Availability

All data sets related to this work are available from the repository: 10.5281/zenodo.13960515.

## Supporting Information Available

Electrochromic activation data; SEM images of WO_3_ with different BiVO_4_ drop-casting cycles; SEM image in backscattered mode and EDX elemental mapping images of Au/BiVO_4_/WO_3_; XPS spectrum of BiVO_4_/WO_3_; table of XPS binding energies of BiVO_4_/WO_3_; absorption spectra of BiVO_4_ and BiVO_4_/WO_3_ series with different BiVO_4_ layers; absorbance as a function of the thickness plot of BiVO_4_ series; table of absorbance and thickness with respect to drop-casting cycles; light-chopped LSV curves of BiVO_4_ and BiVO_4_/WO_3_ series with different BiVO_4_ layers; photostability test of Au/BiVO_4_/WO_3_ and BiVO_4_/WO_3_; and table of comparative PEC results (PDF)
