# Supplementary material for: Integration of Gold Nanoparticles into BiVO4/WO3 Photoanodes via Electrochromic Activation of WO3 for Enhanced Photoelectrochemical Water Splitting
Source: ACS Appl Energy Mater. 2025 Mar 28;8(7):4090–102. doi: 10.1021/acsaem.4c02735 (PMC12001204; doi:10.1021/acsaem.4c02735)
Supplement: Supplementary file 1 — ae4c02735_si_001.pdf [file ae4c02735_si_001.pdf]

Supporting Information

Integration of gold nanoparticles into $BiVO_4/WO_3$ photoanodes via electrochromic activation of WO_3 for enhanced photoelectrochemical water splitting

Ali Can Güler[1,4,*], Milan Masař[1], Michal Urbánek[1], Michal Machovský[1], Mohamed M. Elnagar[2], Radim Beranek[2,*], and Ivo Kuřitka[1,3,*]

[1]Centre of Polymer Systems, Tomas Bata University in Zlin, Tr. T. Bati 5678, 760 01 Zlin, Czech Republic

[2]Institute of Electrochemistry, Ulm University, Albert-Einstein-Allee 47, 89081 Ulm, Germany

[3]Department of Chemistry, Faculty of Technology, Tomas Bata University in Zlín, Vavrečkova 5669, 760 01 Zlín, Czech Republic

[4]Faculty of Chemistry, Jagiellonian University, ul. Gronostajowa 2, Kraków 30-387, Poland

Email addresses: masar@utb.cz; murbanek@utb.cz; machovsky@utb.cz; mohamed.elnagar@uni-ulm.de; *corresponding authors: ali.guler@uj.edu.pl; radim.beranek@uni-ulm.de, kuritka@utb.cz

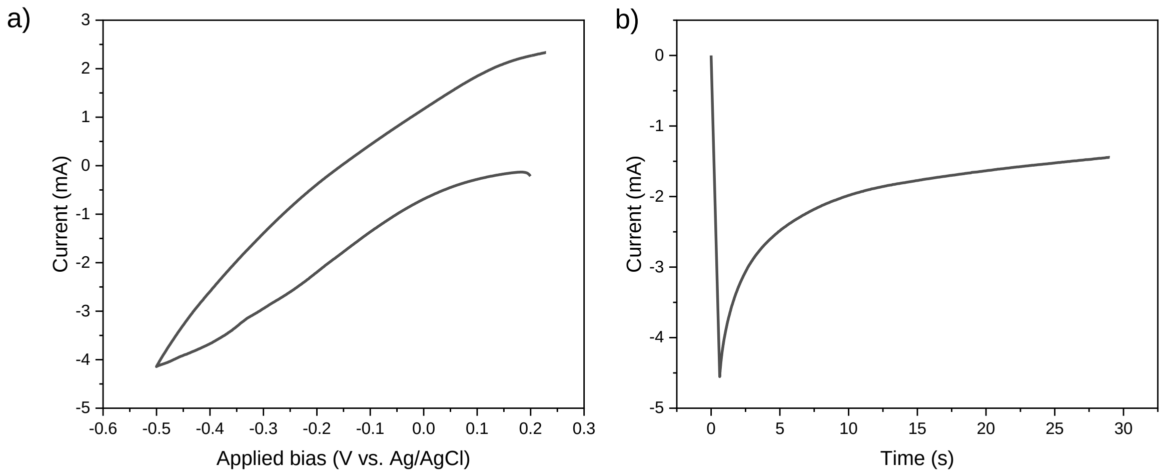

Figure S1. a) Cyclic voltammetry measurement to determine the optimal charging voltage and b) The charging process at –0.4 V for 30 s of $BiVO_4/WO_3$ electrode in 0.5 M H_2SO_4 aqueous solution.

The cyclic voltammogram of $BiVO_4/WO_3$ electrode in 0.5 M H_2SO_4 aqueous solution in the potential range from +0.2 V to –0.5 V *vs*. Ag/AgCl is presented in **Figure S1a**. The current steadily increased up to –4 mA, referring to the electrochromic activation of $BiVO_4/WO_3$. On the other hand, the charging process of $BiVO_4/WO_3$ is depicted in **Figure S1b**. Initially, the observed current reached up to –4 mA, then rapidly elevated to stabilize around –2 mA, indicating that most of the charging process was completed within the first 10 s. To achieve a stable charging current, the process continued for a total duration of 30 seconds

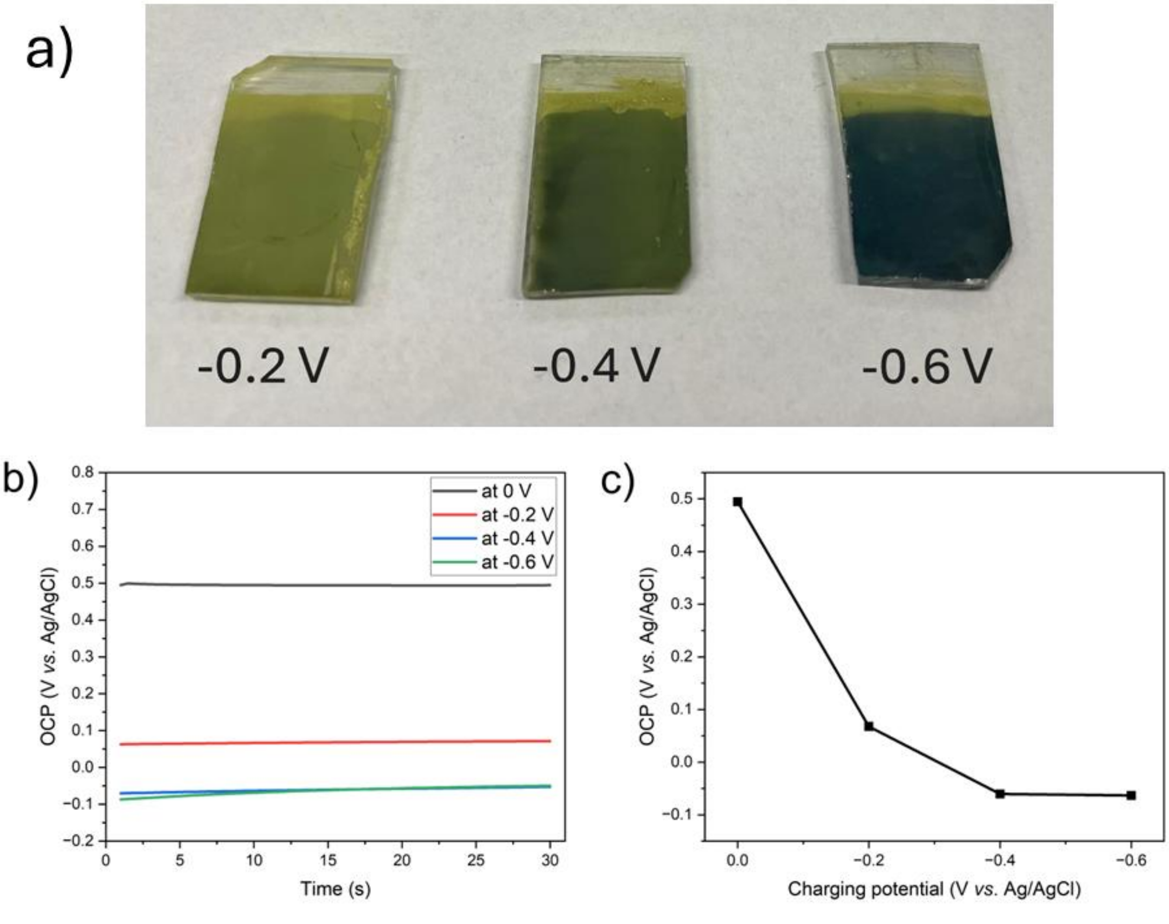

Figure S2. a) Optical images and b) Open-circuit potential measurements in the dark of the charged $BiVO_4/WO_3$ heterostructure thin films at different charging potentials, and c) Variation of the open circuit potential as a function of charging potential.

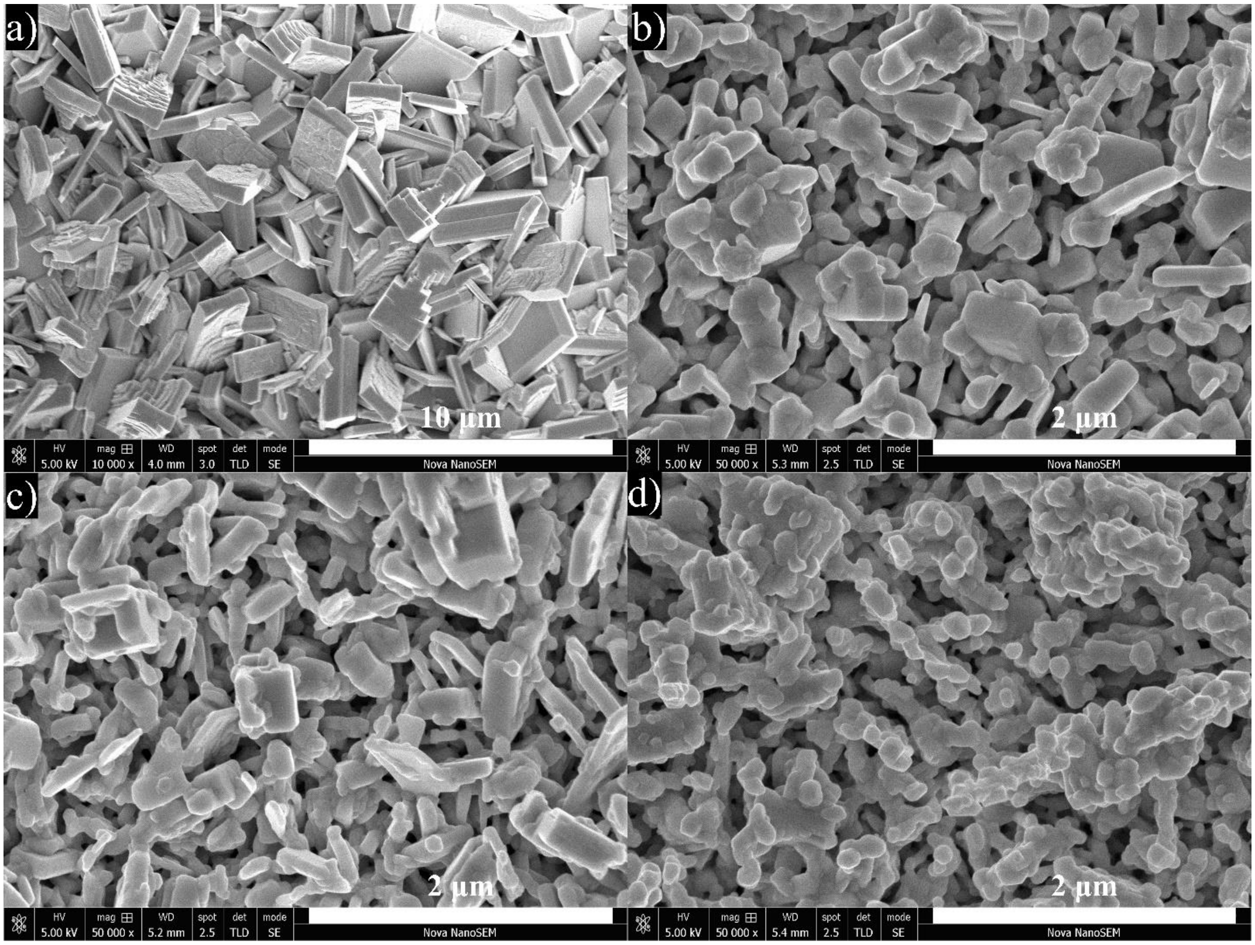

Figure S3. SEM image of a) The as-grown WO_3, b) $BiVO_4$-1c/WO_3, c) $BiVO_4$-2c/WO_3, and d) $BiVO_4$-3c/WO_3 heterostructures.

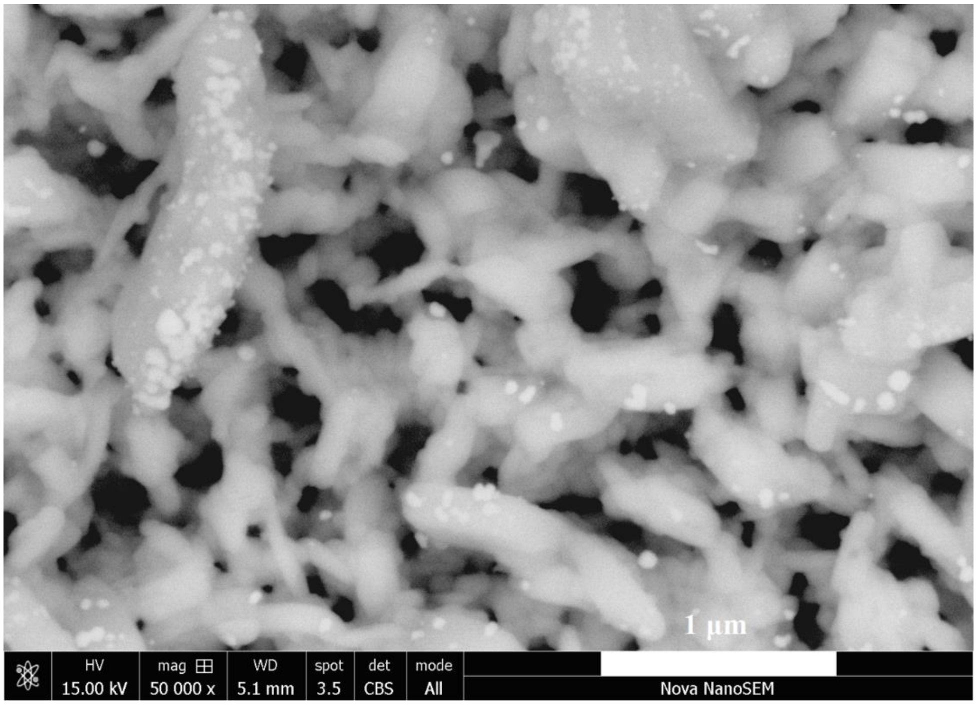

Figure S4. SEM image of $Au/BiVO_4/WO_3$ photoanode in backscattered mode.

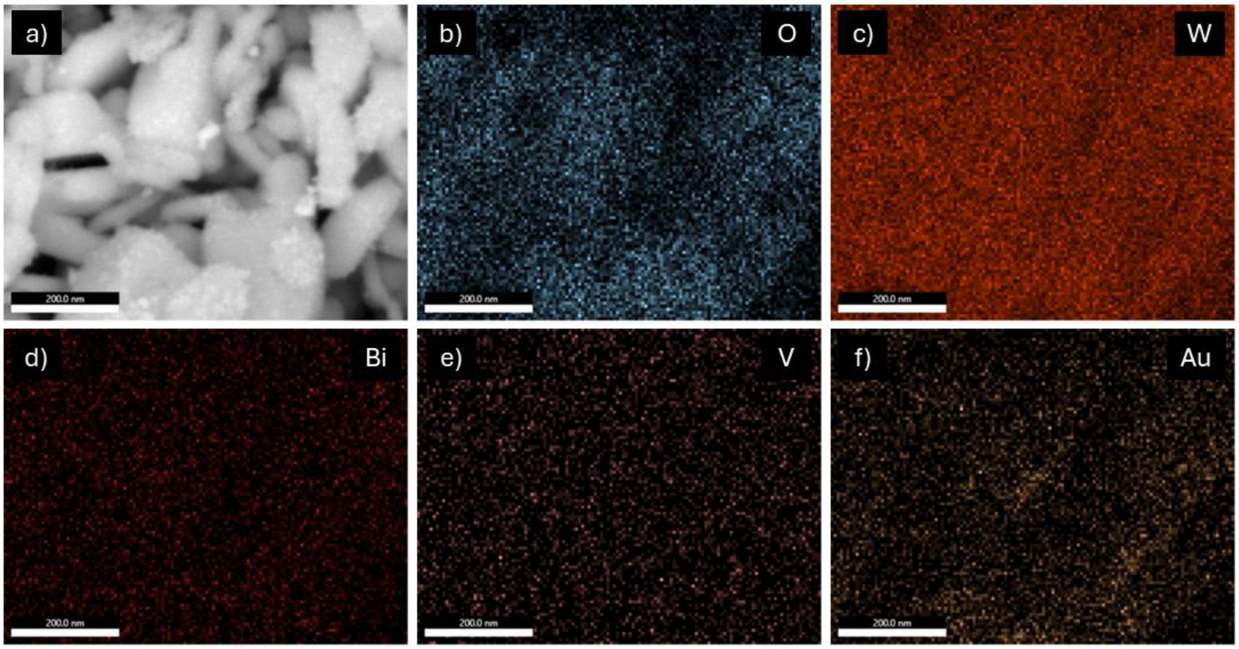

Figure S5. a) SEM image and b-f) Corresponding EDX elemental mapping images of $Au/BiVO_4/WO_3$ photoanode.

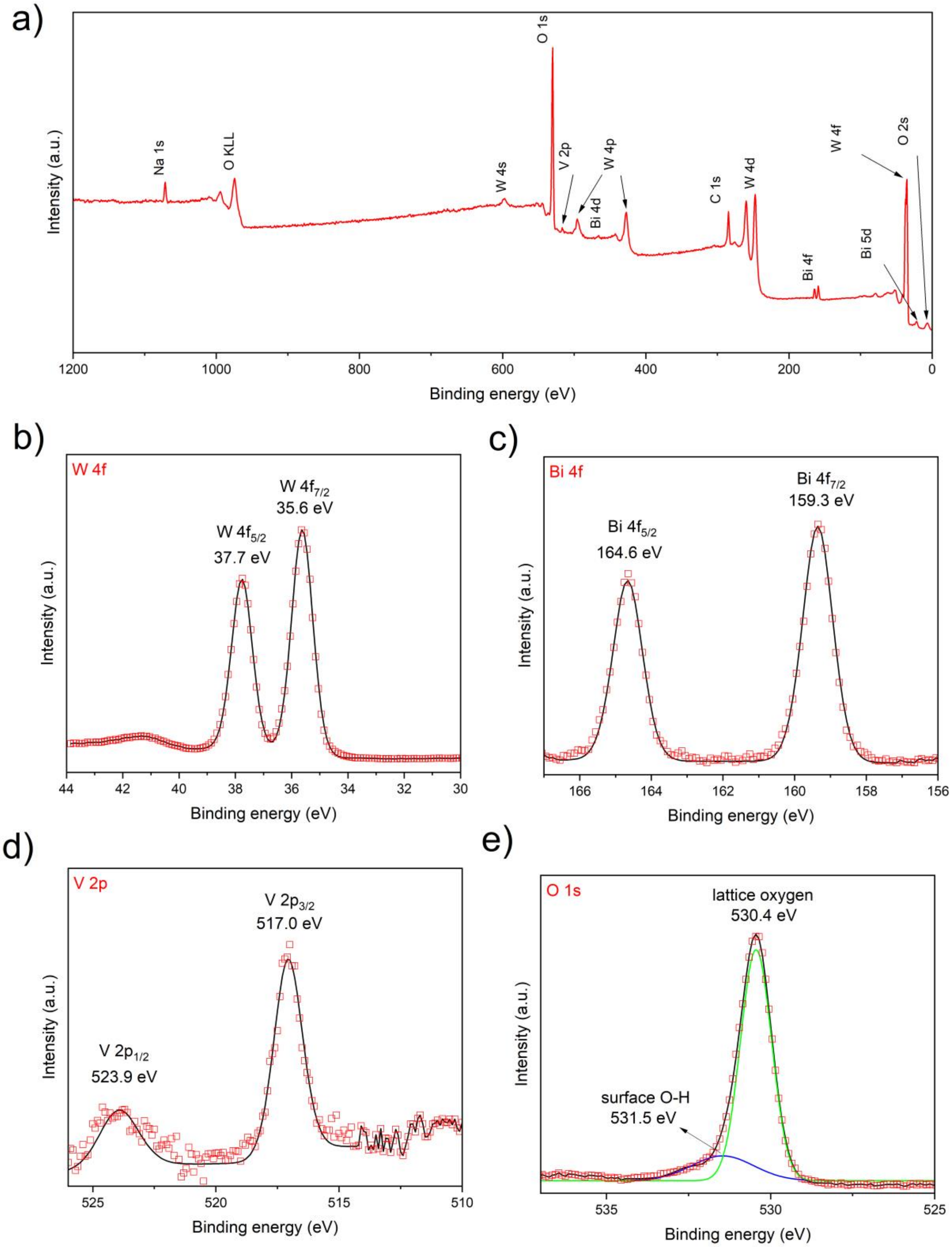

Figure S6. a) The XPS survey spectrum of $BiVO_4/WO_3$, and High-resolution XPS spectra of b) W 4f, c) Bi 4f, d) V 2p, and e) O 1s regions.

Table S1. Comparison of the XPS binding energies.

XPS core-level	Peak position for $BiVO_4/WO_3$ (eV)	Peak positions for $Au/BiVO_4/WO_3$ (eV)
W $4f_{5/2}$	37.7	37.7
W $4f_{7/2}$	35.6	35.5
Bi $4f_{5/2}$	164.6	164.6
Bi $4f_{7/2}$	159.3	159.3
V $2p_{1/2}$	523.9	524.4
V $2p_{3/2}$	517.0	516.9
Au $4f_{5/2}$	-	87.6
Au $4f_{7/2}$	-	83.9
O 1s (lattice oxygen)	530.4	530.2
O 1s (surface O-H)	531.5	531.5

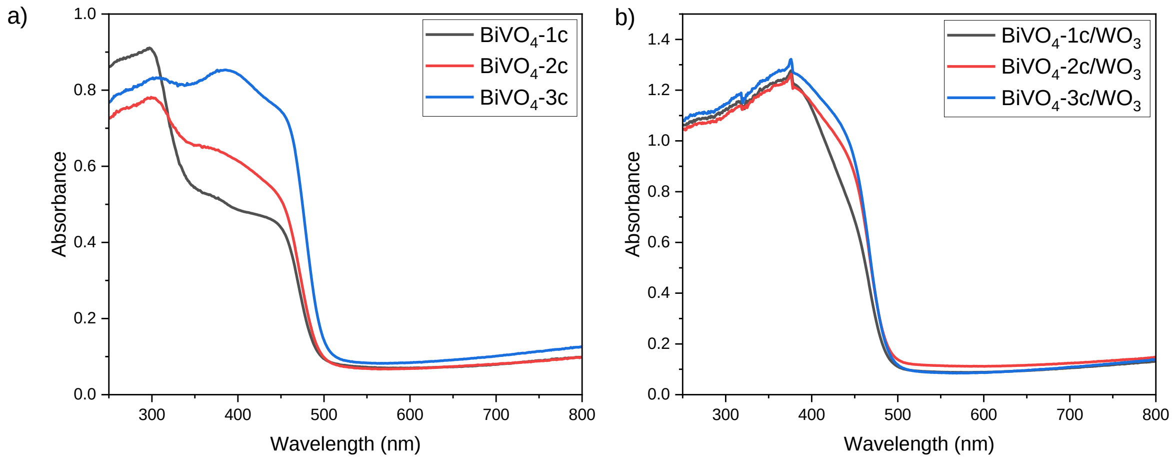

Figure S7. Absorption spectra of a) the $BiVO_4$ series and b) the $BiVO_4/WO_3$ series with different number of $BiVO_4$ layers (1-3).

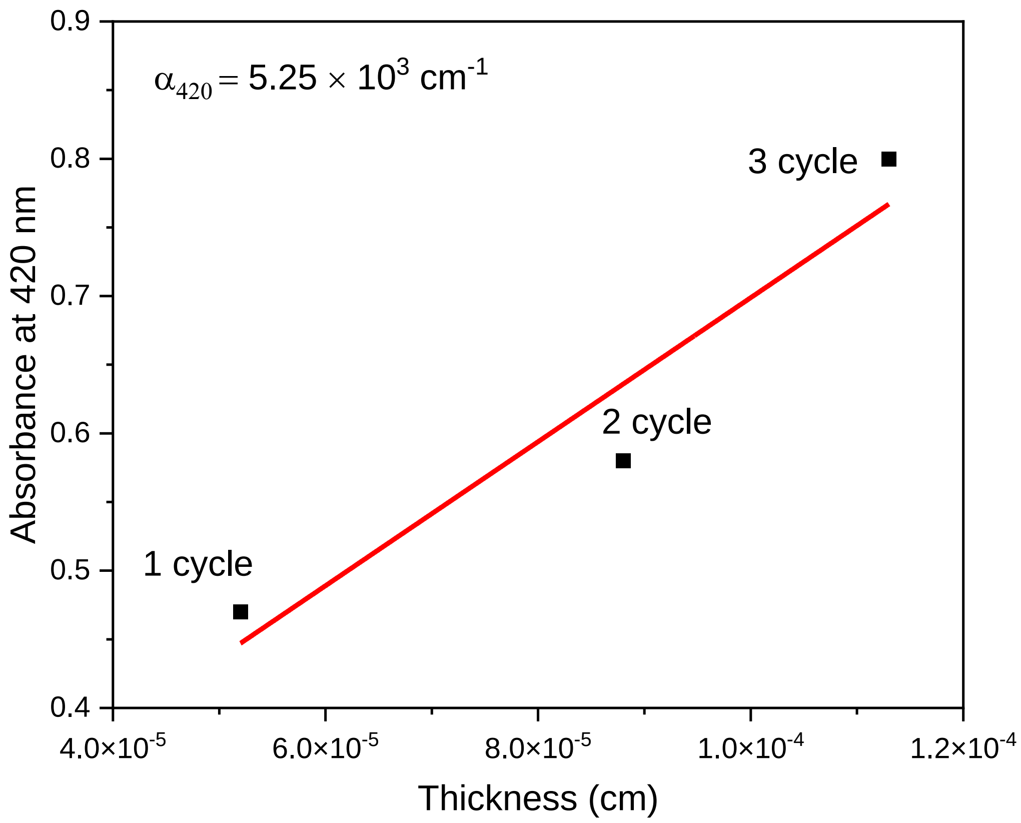

Figure S8. Absorbance (at 420 nm)-thickness plot of $BiVO_4$ thin films prepared by the consecutive depositions of one, two and three $BiVO_4$ layers.

Table S2. Absorbance at 420 nm and thickness of the $BiVO_4$ on WO_3 photoanode upon different number of drop-casting steps.

Drop-casting cycles	Absorbance at 420 nm	Rough Thickness (nm)
1	0.37	705
2	0.46	877
3	0.54	1029

As shown in **Figure S9a**, $BiVO_4$-2c produced the highest photocurrent density (0.005 mA cm^{-2} at 1.23 V *vs*. RHE) among all $BiVO_4$ samples prepared with different deposition cycles on FTO. Moreover, **Figure S9b** presents the light-chopped LSV curves of the $BiVO_4/WO_3$ prepared

with different $BiVO_4$ deposition cycles. The photocurrent density attained from $BiVO_4/WO_3$ progressively increased with the increasing loading amount of $BiVO_4$ up to 2 cycles but decreased significantly in the third cycle. In general, photodevices are designed for illumination through the overlayer, thus a trade-off exists between parasitic light absorption and the surface sensitizer to govern the PEC activity. The $BiVO_4$-2/WO_3 was optimized to be the best performing heterostructure with the maximum obtained photocurrent of 0.623 mA cm^{-2} (at 1.23 V *vs.* RHE). The PEC ranking of $BiVO_4/WO_3$ series is in good harmony with their SEM results (Figure S3). To emphasize again, introducing $BiVO_4$ for the third time aggregated the $BiVO_4$ NPs, which occupy the large voids between the WO_3 nanobricks and decrease the electrochemically accessible surface area. The suitable pore structure enables the electrolyte to penetrate the film and improves the PEC efficiency.

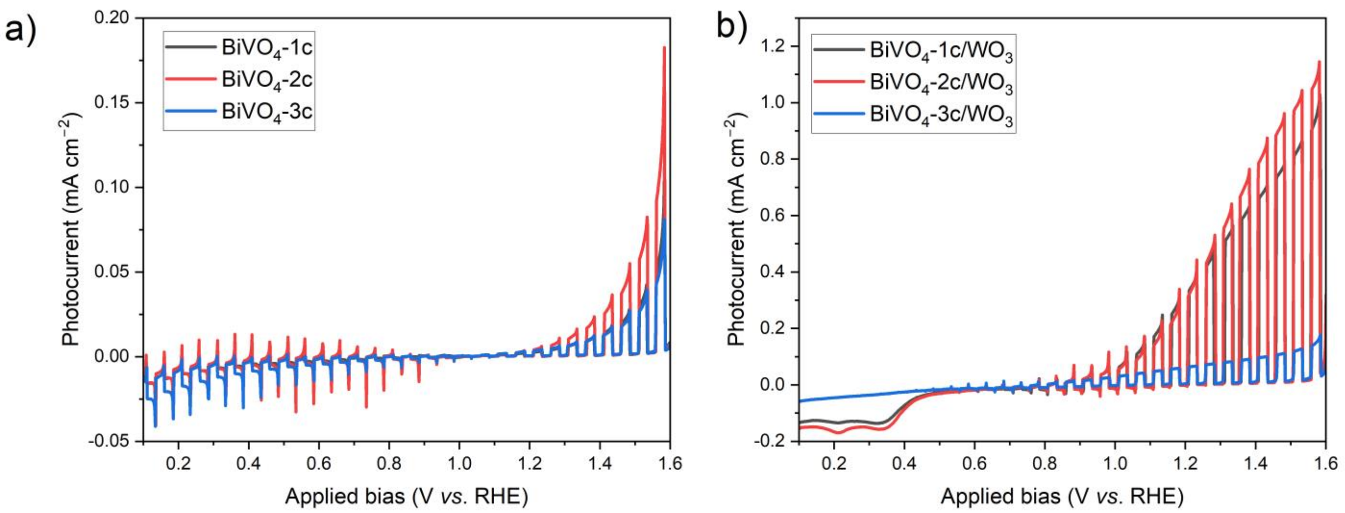

Figure S9. The light-chopped LSV curves under AM 1.5G illumination a) the $BiVO_4$ series and b) the $BiVO_4/WO_3$ series with different cycles of $BiVO_4$ layering.

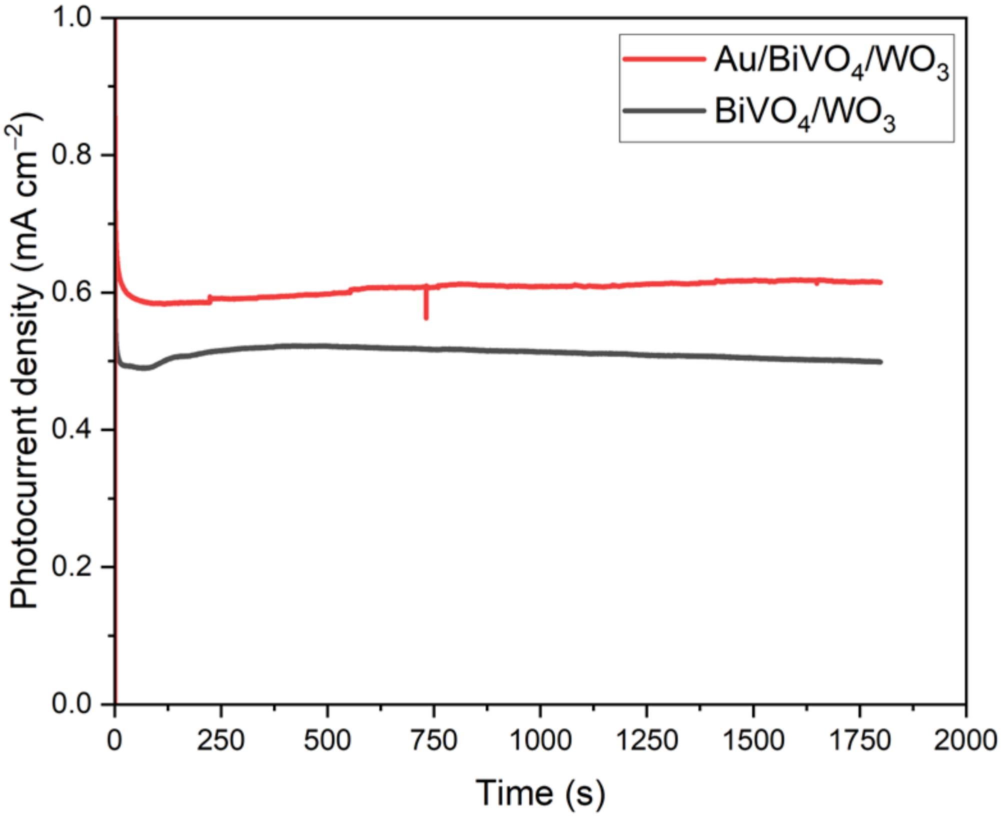

Figure S10. The photostability tests of $BiVO_4/WO_3$ and $Au/BiVO_4/WO_3$ photoanodes for 30 min under AM 1.5G irradiation (87.5 mW cm^{-2}) at 1.23 V *vs*. RHE.

Table S3 compares the PEC water splitting efficiency of $Au/BiVO_4/WO_3$ photoanode with the advanced WO_3-based photoanodes tuned with several techniques such as heterojunction construction with other semiconductors, metal decoration, co-catalyst coupling, or the concurrent combination of these methods. The reported photocurrent density values (at 1.23 V *vs.* RHE) in the previous literature varies in a broad range between 0.16−3.3 mA cm^{-2} depending on the morphological and optical factors, the modification method, and varied experimental parameters by different research groups. As demonstrated (Figure 7b), the photocurrent achieved from $Au/BiVO_4/WO_3$ was effectively increasing with increasing light power under AM 1.5G irradiation. Thus, the direct comparison of the obtained values in this work with those reported in other studies that used AM 1.5G simulated sun light is not straightforward. This is mainly because the simulated light exposure is not highly accurate, particularly in the UV region. Eventually, electrochromic

activation of WO_3 to reduce Au NPs on the optimized $BiVO_4/WO_3$ nanostructure yielded a stable and higher photocurrent among all samples fabricated in this work.

Table S3. Comparative study of the PEC performance of Au/$BiVO_4/WO_3$ photoanode and advanced WO_3-based photoanodes in previous literature.

Heterojunction Photoanode	Modification method	Light source	Electrolyte	Photocurrent density at 1.23 V *vs.* RHE
WO_3 NRs/Co_3O_4 [1]	Electrochemical	LED illumination at ~80 mW cm^{-2}	0.1 M Na_2SO_4 with 0.1 M phosphate buffer at pH 7	~0.16 mA cm^{-2}
WO_3 NPs/Au [2]	Precipitation	A 300 W Xe light at 100 mW cm^{-2}	0.1 M Na_2SO_4 at pH 6.2	~0.6 mA cm^{-2}
WO_3 NRs/$CuWO_4$/CuO [3]	Electrodeposition	A 300 W Xe light at 100 mW cm^{-2}	0.5 M Na_2SO_4	~2.24 mA cm^{-2}
WO_3 planar/$BiVO_4$ facets matching [4]	Spin coating	A 300 W Xe light with AM 1.5G (1 sun)	0.5 M Na_2SO_4	~1 mA cm^{-2}
$WO_3/BiVO_4$/Bi [5]	Continuous electrodeposition	A 100 W Xe light with AM 1.5G at 100 mW cm^{-2}	0.5 M Na_2SO_4 with 0.1 M phosphate buffer at pH 7	~0.7 mA cm^{-2}
WO_3 NRs/$BiVO_4$/Co-Pi [6]	Spin coating	A 300 W Xe light with AM 1.5G at 100 mW cm^{-2}	0.1 M potassium phosphate buffer at pH 7	~3.3 mA cm^{-2}
Au/$BiVO_4/WO_3$/Au [7]	Citrate reduction	Solar simulator at 100 mW cm^{-2}	0.5 M Na_2SO_4 at pH 6.5	~1.31 mA cm^{-2}
Au/$BiVO_4/WO_3$ in this study	Electrochromic activation	Solar simulator with AM 1.5G at 87.5 mW cm^{-2}	0.5 M Na_2SO_4	~0.57 mA cm^{-2}

NRs=nanorods; NPs=nanoplates
